# Molecular Dissection of the *Campylobacter jejuni* CadF and FlpA Virulence Proteins in Binding to Host Cell Fibronectin

**DOI:** 10.3390/microorganisms8030389

**Published:** 2020-03-11

**Authors:** Prabhat K. Talukdar, Nicholas M. Negretti, Kyrah L. Turner, Michael E. Konkel

**Affiliations:** School of Molecular Biosciences, College of Veterinary Medicine, Washington State University, Pullman, WA 99164-7520, USA; prabhat.talukdar@wsu.edu (P.K.T.); nick.negretti@wsu.edu (N.M.N.); kyrah.turner@wsu.edu (K.L.T.)

**Keywords:** pathogenesis, bacteria–host cell interactions, MSCRAMM, fibronectin, adhesion, virulence determinants, effector proteins

## Abstract

*Campylobacter jejuni*, a zoonotic pathogen that frequently colonizes poultry, possesses two Microbial Surface Components Recognizing Adhesive Matrix Molecule(s) (MSCRAMMs) termed CadF and FlpA that bind to the glycoprotein fibronectin (FN). Previous to this study, it was not known whether the CadF and FlpA proteins were functionally redundant or if both were required to potentiate host cell binding and signaling processes. We addressed these questions by generating a complete repertoire of *cadF* and *flpA* mutants and complemented isolates, and performing multiple phenotypic assays. Both CadF and FlpA were found to be necessary for the maximal binding of *C. jejuni* to FN and to host cells. In addition, both CadF and FlpA are required for the delivery of the *C. jejuni* Cia effector proteins into the cytosol of host target cells, which in turn activates the MAPK signaling pathway (Erk 1/2) that is required for the *C. jejuni* invasion of host cells. These data demonstrate the non-redundant and bi-functional nature of these two *C. jejuni* FN-binding proteins. Taken together, the *C. jejuni* CadF and FlpA adhesins facilitate the binding of *C. jejuni* to the host cells, permit delivery of effector proteins into the cytosol of a host target cell, and aid in the rewiring of host cell signaling pathways to alter host cell behavior.

## 1. Introduction

Fibronectin (FN)-binding proteins (FNBPs) are members of the Microbial Surface Components Recognizing Adhesive Matrix Molecule(s) (MSCRAMMs) family. These proteins are bacterial surface proteins that mediate the attachment of bacteria to FN and can promote adhesion to cells. Given that FNBPs frequently contribute in establishing an infection, a significant amount of effort has been put forth to identify and characterize these proteins in both Gram-positive and Gram-negative bacteria. For example, *Staphylococcus aureus* possesses fibronectin-binding protein A (FNBPA), fibronectin-binding protein B (FNBPB), and ClfA [1,2], and *Streptococcus pyogenes* possesses fibronectin-binding protein I (SfbI), fibronectin-binding protein F (SfbF), and M protein [3]. FNBPs have also been identified in Gram-negative pathogens, such as ShdA in *Salmonella enterica* serotype Typhimurium [4], YadA *in Yersinia* spp. [5], BBK32, RevA, and RevB in *Borrelia burgdorferi* [6,7], and TP0136 in *Treponema pallidum* [8]. Defining the role of MSCRAMMs in promoting bacteria–host interactions has provided a better understanding of the process of microbial colonization and disease.

*Campylobacter jejuni* is a Gram-negative, curve-shaped, motile, microaerophilic bacterium. This organism is one of the leading bacterial causes of diarrhea in the United States and accounts for 5–14% of diarrheal cases worldwide [9]. In the United States, there are approximately 1.4–2.3 million *Campylobacter* infections each year [10]. Most cases of the disease result from the handling and consumption of undercooked poultry products and from foods cross-contaminated with raw poultry products. Campylobacteriosis generally occurs 2–5 days after ingestion. *C. jejuni* infected individuals suffer from fever, nausea, malaise, abdominal pain, and loose to watery stools, which may contain blood and/or fecal leukocytes [11]. In addition, infection with particular strains of *C. jejuni* correlates with a higher incidence of Guillain–Barré syndrome (GBS), Miller Fisher syndrome, reactive arthritis, and post-infectious irritable bowel syndrome (PI-IBS) [12,13,14]. *C. jejuni* is the most common antecedent to GBS. The cost associated with treating acute *C. jejuni* infections and GBS in the U.S. is estimated to be $1.2 billion per year [14]. Generally, *C. jejuni* infection occurs when the motile bacterium attaches to, invades, and propagates within host intestinal tissues, which results in blood and mucus in the stool [11,15]. Scientists have identified several *C. jejuni* adhesins that play a critical role in disease symptoms and progression [16,17]. However, further research is necessary to understand *Campylobacter* virulence and to develop novel therapeutic and immune augmentation strategies that overcome the shortcomings inherent with traditional antibiotics.

*C. jejuni* possesses at least two outer membrane-embedded FNBPs, termed CadF for *Campylobacter* adhesion to fibronectin and FlpA for fibronectin-like protein A (FlpA) [16,17]. The *cadF* gene, which is 960 bp and located first in a bicistronic operon, encodes a 37 kDa protein [16]. The protein possesses a signal peptide sequence, an outer membrane (OM) channel superfamily domain, and an outer membrane protein A (OmpA)-like domain. The *flpA* gene, which is 1236 bp and located second in a polycistronic operon containing three genes, encodes a 46 kDa protein [18]. The FlpA protein contains a signal peptide sequence and three FN-type III domains. Recombinant CadF and FlpA proteins have been purified and demonstrated to bind to FN in a dose-dependent and saturable fashion [18,19]. CadF mediates binding to FN via a four amino acid motif (Phe-Arg-Leu-Ser) [20], whereas the FN-binding site within FlpA has been localized to a span of nine amino acids (Trp-Arg-Pro-His-Pro-Asp-Phe-Arg-Val) [19]. As mentioned above, disruption of either *cadF* or *flpA* results in *C. jejuni* mutants impaired in their ability to bind to cultured epithelial cells [16,18]. Moreover, *C. jejuni cadF* and *flpA* deletion mutants demonstrate a marked impairment in their ability to colonize chickens [17,21]. Studies have yet to be performed to examine the phenotypic properties (cell adherence and FN-binding) of *cadF* or *flpA* expressed singly in a *C. jejuni cadF flpA* double deletion mutant.

While advances have been made in understanding the biology of *C. jejuni*, genetic manipulation of this bacterium remains challenging. Perhaps the most significant technical obstacle has been in the functional complementation of specific genes. Possible reasons for the lack of functional complementation include aberrant levels of gene expression and protein synthesis due to inappropriate gene copy numbers or alterations in the topological characteristics of the DNA. It has become common for researchers to insert a wild-type copy of the gene into the chromosome (*in cis*) for complementation. Karlyshev and Wren [22] published an efficient method for gene insertion into the *C. jejuni* chromosome, whereby the gene of interest is inserted into an rRNA gene cluster by homologous recombination. The benefits of this method are that 1) only one suicide vector needs to be generated for use with multiple *C. jejuni* strains, as the rRNA gene sequences are conserved among strains and 2) there is little to no effect on bacterial growth and cell function unless the cloned gene itself has a deleterious effect.

Genetic redundancy is where two or more genes encode proteins with the same apparent function. A widespread view is that such redundancy is not evolutionarily stable. However, in the instance where both proteins function with high efficiency or the two proteins interact with the same target but have different functional outcomes, genetic redundancy is evolutionarily stable. The question that arises when two genes encode proteins with similar functions is whether a given protein is ‘necessary’ or ‘sufficient’ for a phenotype. Although ample evidence exists that CadF and FlpA are FNBPs and bona fide adhesins, it has been challenging to address the contribution of each protein individually. The obstacle in addressing the contribution of CadF or FlpA is that it requires the complementation of a *cadF flpA* double deletion mutant with *cadF*, *flpA*, or both genes. More specifically, it has not been possible to generate a complementation vector harboring the *cadF* gene because the expression of the entire *cadF* coding sequence in *E. coli* is toxic. Here we describe a novel approach for the complementation of the *cadF* gene. We utilized this method to complement a *C. jejuni cadF flpA* double deletion mutant with *cadF*, *flpA*, and both *cadF* and *flpA* to assess CadF and FlpA function in the context of a viable bacterium. We believe that the genetic approach described herein can be applied to other *C. jejuni* genes that have been challenging to complement, as well as to other bacteria where *E. coli* is used as an intermediate host to generate the vector for functional complementation studies. In a broader context, the findings from this study are widely applicable to understanding disease regulation by FNBPs present in other Gram-positive and Gram-negative bacteria.

## 2. Materials and Methods

### 2.1. Bacterial Strains and Growth Conditions

A complete list of the *C. jejuni* isolates used in this study is provided in Table 1. The *C. jejuni* 81-176 wild-type strain was grown on Mueller–Hinton (MH) agar plates supplemented with 5% citrated bovine blood (MH-blood) and 2 μg/mL of tetracycline (Tet) or in MH broth with tetracycline in a microaerobic (5% O_2_, 10% CO_2_, 85% N_2_) atmosphere at 37 °C. *C. jejuni* mutants and complemented isolates were selected on MH blood agar plates with appropriate antibiotics: chloramphenicol (Cm, 8 μg/mL), hygromycin B (Hygro, 250 μg/mL), kanamycin sulfate (Kan, 50 μg/mL), spectinomycin (Spec, 200 μg/mL), and tetracycline (Tet, 2 μg/mL). All cultures were passaged every 24 to 48 h. *Escherichia coli* Stellar™ cells were maintained on Luria–Bertani (LB) agar plates or in LB broth aerobically at 37 °C. For chemical transformation, *E. coli* competent cells were grown in SOC medium before plating on LB agar plates supplemented with appropriate antibiotics: chloramphenicol (Cm, 20 μg/mL), hygromycin B (Hygro, 250 μg/mL), kanamycin sulfate (Kan, 50 μg/mL), and spectinomycin (Spec, 100 μg/mL).

### 2.2. Generation of C. jejuni 81-176 cadF, flpA and cadF flpA Deletion Mutants

All plasmids used in this study are listed in Table 2. The primers used in this study are listed in Table 3. A *C. jejuni* 81-176 *cadF* deletion mutant (Δ*cadF*) was generated by homologous recombination using a suicide vector harboring a deleted copy of the *cadF* gene. The 5′ and 3′ flanking regions of the *cadF* gene were PCR amplified with primer pairs MEK4067/MEK4068 and MEK4063/MEK4064, respectively. The chloramphenicol resistance cassette (CAT) was PCR amplified from the *C. jejuni*–*E. coli* pRY111 shuttle vector with the MEK4065 and MEK4066 primers. The 5′ *cadF* flanking region, chloramphenicol resistance cassette, and 3′ *cadF* flanking region were cloned into the suicide plasmid pBSK-Kan2 using the Clontech In-Fusion HD cloning kit (Takara Bio USA, Inc., Mountain View, CA, USA), resulting in the plasmid pBSK-Kan2-*cadF-*CAT. This suicide vector was electroporated into the *C. jejuni* 81-176 wild-type strain, and chloramphenicol-resistant (Cm^R^) transformants were selected on MH blood agar plates supplemented by chloramphenicol.

To generate a *C. jejuni* 81-176 *flpA* deletion mutant (Δ*flpA*), another suicide vector was created that harbors the 5′ flanking region of the *flpA* gene followed by a kanamycin resistance cassette (Kan2) and the 3′ flanking region of *flpA*. The 5′ and 3′ flanking regions of the *flpA* gene were PCR amplified with primer pairs MEK4073/MEK4074 and MEK4069/MEK4070, respectively. The *aphA-3* gene encoding Kan2 was PCR amplified from the pBSK-Kan2 suicide vector with the MEK4071 and MEK4072 primers. In-fusion cloning was used to clone these three DNA fragments into the suicide plasmid pBSK, resulting in the pBSK-*flpA-*Kan2 plasmid. This suicide vector was electroporated into the *C. jejuni* 81-176 wild-type strain, and kanamycin-resistant (Kan^R^) transformants were selected on a MH blood agar plate supplemented with kanamycin. The pBSK-*flpA-*Kan2 plasmid was also incorporated into the *C. jejuni* 81-176 Δ*cadF* mutant to generate a *C. jejuni* 81-176 *cadF flpA* double deletion mutant (Δ*cadF* Δ*flpA*). The *C. jejuni* Δ*cadF*, Δ*flpA*, and Δ*cadF* Δ*flpA* mutants were confirmed by PCR using gene-specific primers.

### 2.3. Generation of C. jejuni cadF Complemented Isolates

The complementation of the Δ*cadF* mutant was achieved using a two-step electroporation with a series of different constructs. The first construct that was generated contained the promoterless *cadF* gene fused to a FLAG-tag. Briefly, the *cadF* coding sequence was PCR amplified from the *C. jejuni* 81-176 wild-type strain with the MEK4507 and MEK4508 primers. The amplified product was cloned between the 16S rRNA and 23S rRNA genes contained in the prRNA-Hygro^R^ suicide vector using in-fusion cloning, resulting in the prRNA-Hygro^R^-*cadF*_NP_-FLAG vector. This vector was electroporated into the Δ*cadF* mutant, resulting in a Δ*cadF*::promoterless *cadF*-FLAG-tag isolate (Δ*cadF*::*cadF*_NP_-FLAG isolate). All transformants were selected on MH blood agar plates supplemented with hygromycin B and confirmed by PCR.

This Δ*cadF*::*cadF*_NP_-FLAG isolate was electroporated a second time with one of the three constructs generated below. The three different constructs contained various lengths of the *cadF* 5′ coding sequence and were each generated using in-fusion cloning of three individual fragments. The first (variable) fragment contained the native *cadF* promoter of 154 bp and either 283 bp, 334 bp, or 400 bp of the *cadF* gene (*P_cadF_ cadF*_283 bp_, *P_cadF_ cadF*_334 bp_, and *P_cadF_ cadF*_400 bp_) and was generated by PCR amplification using primers MEK4541/MEK4542 (437 bp), MEK4541/MEK4543 (488 bp), or MEK4541/MEK4544 (554 bp), respectively. The second fragment contained the *aad9* spectinomycin resistance gene [27] driven by the *C. jejuni hupB* promoter (gBlocks Gene Fragment, Integrated DNA Technologies, San Diego, CA, USA) and was amplified with the MEK4509 and MEK4518 primers. The third fragment contained the 23S rRNA gene and was PCR amplified from the prRNA-Hygro^R^ vector. The final constructs were designated prRNA-Spec^R^-*P_cadF_ cadF*_400 bp_, prRNA-Spec^R^-*P_cadF_ cadF*_334 bp_, and prRNA-Spec^R^-*P_cadF_ cadF*_283 bp_, respectively. These three plasmids were individually transformed into the *C. jejuni* Δ*cadF*::*cadF*_NP_-FLAG isolate by electroporation, and spectinomycin-resistant (Spec^R^) transformants were selected on MH blood agar plates supplemented with spectinomycin. All three electroporations resulted in individual transformants that possessed a wild-type copy of the *cadF* gene expressed from its native promoter (Δ*cadF*::*P_cadF_*_-400 bp_ *cadF*-FLAG, Δ*cadF*::*P_cadF_*_-334 bp_ *cadF*-FLAG, Δ*cadF*::*P_cadF_*_-283 bp_ *cadF*-FLAG isolates). A similar strategy was used to complement the Δ*cadF* Δ*flpA* double mutant, where the prRNA-Hygro^R^-*cadF*_NP_-FLAG construct was used to generate the intermediate *C. jejuni* Δ*cadF* Δ*flpA*::*cadF*_NP_-FLAG isolate. This intermediate isolate was used for the incorporation of vectors prRNA-Spec^R^-*P_cadF_ cadF*_400 bp_, prRNA-Spec^R^-*P_cadF_ cadF*_334 bp_, and prRNA-Spec^R^-*P_cadF_ cadF*_283 bp_ to generate *C. jejuni* Δ*cadF* Δ*flpA*:: *P_cadF_* *cadF*-FLAG isolates.

### 2.4. Generation of C. jejuni flpA Complemented Isolates

Two different constructs containing the *flpA* gene and promoter were used to functionally complement *C. jejuni* mutants. The prRNA-Hygro^R^-*P_flpA_ flpA* suicide vector was introduced into the Δ*flpA* mutant and Δ*cadF* Δ*flpA* double mutant. Briefly, a 1459 bp DNA fragment containing the *flpA* gene was PCR amplified from the *C. jejuni* 81-176 wild-type strain with the MEK4602 and MEK4603 primers. This fragment contained the *flpA* gene (1236 bp) and the *flpA* promoter sequence (223 bp). The fragment was cloned in between the 16S and 23S rRNA genes at the XbaI-BamHI site of the prRNA-Hygro^R^ vector, resulting in the plasmid prRNA-Hygro^R^-P*_flpA_ flpA*. This suicide vector was introduced into the mutants by electroporation, and transformants were selected on MH blood agar plates supplemented with hygromycin B. All Hygro^R^ transformants were determined to contain the *flpA* gene by PCR using gene-specific primers.

The prRNA-Spec^R^-*P_flpA_ flpA*-*P_cadF_ cadF*_400 bp_ suicide vector was introduced into the Δ*cadF* Δ*flpA* double mutant. Briefly, the *flpA* gene and promoter were amplified by PCR from *C. jejuni* 81-176 wild-type strain with the MEK4676 and MEK4677 primers. The fragment was cloned into the SacII site located between *cadF* promoter and spectinomycin resistance cassette of the prRNA-Spec^R^-*P_cadF_ cadF*_400 bp_ vector, generating the plasmid prRNA-Spec^R^-*P_flpA_ flpA*-*P_cadF_ cadF*_400 bp_. The vector was electroporated into the *C. jejuni* Δ*cadF* Δ*flpA*::*cadF*_NP_-FLAG isolate, and transformants were selected on MH blood agar plates supplemented with spectinomycin.

### 2.5. Electroporation and Strain Confirmation (PCR and Sequencing)

The electroporations were conducted using a Bio-Rad *E. coli* Pulser (Bio-Rad, Hercules, CA, USA) and 2 mm gap cuvettes at the 2.5 kV voltage setting. Following electroporation, bacterial suspensions were spread onto MH-blood agar plates for 24 h. Transformants were isolated by streaking the bacterial cells onto MH blood agar plates containing the appropriate antibiotic. The resultant colonies were picked and confirmed by PCR amplification using gene-specific primers.

### 2.6. Gel Electrophoresis and Immunoblotting

Bacterial whole-cell lysates (WCL; OD_540_ equivalent = 0.1 per lane) were separated in SDS 12.5% polyacrylamide gel electrophoresis (PAGE) gels using the discontinuous buffer system described by Laemmli [28]. The proteins were either stained with Coomassie Brilliant Blue R-250 (CBB-R250) or electrophoretically transferred to polyvinylidene fluoride membranes (PVDF; Immobilon P; Millipore Corp., Bedford, MA, USA). The blots were incubated with either a rabbit α-CadF serum (1:4000, K6928) or a rabbit α-FlpA serum (1:4000, L0850) in Tris-buffered saline with 0.1% Tween 20 (TBS-T) containing 9% nonfat dry milk (NFDM). Bound antibodies were detected with peroxidase-conjugated goat α-rabbit immunoglobulin G (Sigma-Aldrich, St. Louis, MO, USA). Immunoblot development was done by chemiluminescence (ECL Prime, GE Healthcare Biosciences, Piscataway, NJ, USA), and reactive proteins were visualized with a GE LAS-4000 mini (GE Healthcare).

### 2.7. Preparation of Outer Membrane Proteins

Outer membrane protein (OMP) extracts were prepared using *N*-lauroylsarcosine (Sigma-Aldrich), as outlined previously [29]. The extracts were washed with 50 mM Tris (pH 7.5), suspended in the same buffer, and stored at −20 °C. The protein concentration of each OMP extract was determined by the bicinchoninic acid (BCA) assay.

### 2.8. Adherence Assay

A stock culture of INT 407 cells (ATCC CCL 6) was cultured in Minimal Essential Media (MEM, Gibco, Grand Island, NY, USA) supplemented with 10% fetal bovine serum (FBS, Premium Grade FBS, 100% US Origin, VWR, Product No. 97068-085) and 1 mM sodium pyruvate (Corning Inc., Manassas, VA, USA) at 37 °C in a 5% CO_2_ incubator. Adherence assays were performed as outlined elsewhere [30], with one modification—the bacteria were suspended in MEM without FBS. All assays in this study were performed at a multiplicity of infection (MOI) ranging between 50 and 500 to one to ensure reproducibility and repeated a minimum of three times. Regardless of the MOI, the trends of the isolates relative to one another were the same.

### 2.9. Biotinylation of FN and FN-Binding Assay

Human FN (Sigma-Aldrich) was suspended in 1 mL phosphate buffered saline (PBS) to a concentration of 5 µM, and biotinylated by adding 1 mg of Sulfo-NHS-LC-Biotin for 3 h at room temperature. Excess biotin was removed using a Zeba desalting column (40k MWCO, Thermo Fisher Scientific, Waltham, MA, USA) and dialyzing overnight in PBS (40k MWCO) at 4 °C. The *C. jejuni* isolates were grown overnight in MH broth, pelleted, and resuspended to an OD_540_ = 0.3 in PBS (~3 × 10^8^ CFU/mL). A suspension containing 80 µL of bacteria in PBS and 20 µL of biotinylated FN was mixed, incubated for 1 h at room temperature, and *C. jejuni* pelleted by centrifugation. The amount of biotinylated FN in the supernatant was determined using a Pierce Biotin Quantitation Kit, as outlined by the manufacturer (Thermo Fisher Scientific).

### 2.10. Enzyme-Linked Immunosorbent Assays (ELISAs) with CadF and FlpA Peptides

CadF, FlpA, and scrambled peptides were chemically synthesized by GenMed Synthesis Inc. (San Antonio, TX, USA) and were made up of an N-terminal Nano-tag [31] and 28 amino acids of the respective proteins. The peptide sequences are:

(1) CadF: DVEAWLGARVPLVETSGGFGHYGAGVK**FRLS**DSLALRLETRDQ;

(2) FlpA: DVEAWLGARVPLVETNRIKLI**WRPHPDFRV**DSYIIERTKGDDK; and

(3) Scrambled: DVEAWLGARVPLVETPGLGALKDSGHHDGDLSIRSRRFFPRDH,

where the residues in bold highlight the FN-binding domains. To assess FN-binding, 1 µg/well of each peptide was used to coat the well of a polycarbonate 96-well tray in a 0.1 M carbonate buffer (pH 9.5) overnight at 4 °C. Wells were then washed with PBS and blocked with 0.1% bovine serum albumin (BSA) in PBS for 2 h. Wells were washed with PBS, and a two-fold dilution series of FN was added to the wells ranging from 100 to 1.5625 µg/well. FN was either dissolved in PBS alone or in PBS containing 4 M urea. The plate was incubated overnight at 4 °C. In the experiments testing different FN domains, either the 45 kDa fragment (Cat# F0162, Sigma-Aldrich) or the 30 kDa fragment (Cat# F9911, Sigma-Aldrich) was added in place of whole FN in PBS. Wells were washed and probed with α-FN antibody (Cat# F3648, 1:1000, Sigma-Aldrich) in PBS with 0.1% BSA for 2 h at room temperature. The wells were washed, and a secondary α-rabbit HRP antibody (Cat# A6154, 1:4000, Sigma-Aldrich) in PBS with 0.1% BSA was added for an additional 2 h. After extensive washing, TMB substrate (ThermoFisher Scientific) was added to the wells and incubated for approximately 25 min before quenching with 2.5 M H_2_SO_4_. Trays were read at 450 nm in a BioTek ELx808IU plate reader (BioTek Instruments Inc., Winooski, VT, USA). All samples were assayed three times on independent days and blanked against wells that were coated with BSA. Because the data were combined from independent assays, absorbance is reported as a percentage of relative absorbance where 0% represents the blank value for a well, and 100% represents the maximum value in a well.

### 2.11. Immunoblot Analysis of Total-Erk 1/2 and Phospho-Erk 1/2 in C. jejuni Infected INT 407 Cells

The level of total-Erk 1/2 and phospho-Erk 1/2 in *C. jejuni* infected INT 407 cells was determined by immunoblot analysis. Briefly, INT 407 cells were seeded at a density of 1 × 10^5^ in 24-well tissue culture trays (BD Falcon, Franklin Lakes, NJ, USA, Cat # 353047). The following day, the cells were serum-starved for 4 h. Each well was infected with *C. jejuni* and incubated for 60 min. Following incubation, the media was removed, INT 407 cells were lysed using 2× sample buffer, and lysates were collected and analyzed by SDS-PAGE coupled with immunoblot analysis. The proteins were transferred to a PVDF membrane and probed with a total-Erk 1/2 antibody (Santa Cruz, Dallas, TX, USA, Cat # sc-94) at a 1:500 dilution and a phospho-Erk 1/2 antibody (Cell Signaling Technology, Danvers, MA, USA, Cat # 4377) at a 1:1000 dilution. Following overnight incubation at 4 °C, the blots were rinsed and incubated for 1 h with horseradish peroxidase-conjugated α-rabbit IgG or α-mouse IgG (Sigma) secondary antibodies at a 1:2000 dilution. Blots were developed using ECL prime reagent (GE Healthcare) and imaged using a LAS 4000 mini (GE healthcare). Bands were quantified using ImageJ. Densitometry analysis was performed to determine the ratio of phospho-protein to total protein (i.e., p-Erk 1/2 to total Erk 1/2). Four negative controls were used in all assays: (1) uninfected cells; (2) cells infected with a *C. jejuni ciaD* deletion mutant (defective in secretion of the CiaD effector protein from the flagellum); (3) cells infected with a *C. jejuni flgL* deletion mutant (non-motile, defective in protein secretion from the flagellum); and (4) cells treated with PD98059 (an Erk 1/2 inhibitor). A 50 mM stock solution of Erk 1/2 inhibitor PD98059 (Selleck, Houston, TX, USA, Cat # S1177) was prepared in dimethyl sulfoxide (DMSO) and used at a final concentration of 50 μM. This inhibitor was added to the INT 407 cells 30 min prior to infection and maintained throughout the assay. No significant death of INT 407 cells was observed with the PD98059 treatment, as judged by trypan blue staining (not shown).

### 2.12. Adenylate Cyclase Domain Reporter Delivery Assays

The *ciaD*-*ACD* shuttle plasmid harbors a copy of the *ciaD* gene fused to the adenylate cyclase domain (ACD) of the *Bordetella pertussis* CyaA protein and is expressed from the *C. jejuni cysM* promoter. The CysM protein, which is a 32.4 kDa cytoplasmic protein (*O*-acetylserine sulfhydrylase B) involved in cysteine biosynthesis, is constitutively synthesized in *C. jejuni* [32]. The *ciaD-ACD* shuttle plasmid, used previously [33], was modified by replacing the chloramphenicol resistance gene with the hygromycin resistance gene using the MEK4721 and MEK4722 primers and in-fusion cloning, generating the plasmid pRY111-Hygro^R^-*P_cysM_ ciaD*-*ACD*. This modification was needed, as some of the *C. jejuni* isolates used were chloramphenicol resistant and required a different antibiotic resistance for selection. The *ciaD*-*ACD* shuttle plasmid (pRY111-Hygro^R^-*P_cysM_ ciaD*-*ACD*) was transformed into the *C. jejuni* wild-type strain, Δ*cadF* mutant, Δ*flpA* mutant, Δ*cadF* Δ*flpA* mutant, and Δ*flgL* mutant by conjugation [33]. SDS-PAGE and immunoblots were performed with a rabbit CyaA IgG polyclonal antibody (sc-33620, Santa Cruz Biotechnology) to confirm the *C. jejuni* transformants. The ACD delivery assay was then performed with INT 407 cells, as outlined elsewhere with modifications [33]. Briefly, INT 407 cells were seeded into each well of a 6-well tissue culture tray at 1.6 × 10^6^ cells/well. The following day, the cells were rinsed one time with tissue culture medium and infected with a 3 mL suspension of *C. jejuni* (OD_540_ = 0.3–0.35) in 1% FBS-MEM. The trays were centrifuged for 5 min at 600× *g* to promote bacteria–host cell contact followed by incubation for 45 min at 37 °C in a humidified, 5% CO_2_ incubator. After incubation, the cells were lysed with 0.1 M HCl and 0.1% Triton X-100. Cell lysates were centrifuged for 5 min at 13,000× *g*, and the clarified supernatants were collected in separate tubes. The cAMP level for each sample was assessed using the Direct cAMP ELISA kit (Enzo Life Sciences, Farmingdale, NY, USA) according to the manufacturer’s specifications. Each isolate was tested in biological triplicate on different days for the delivery of the CiaD-ACD fusion protein into the cytosol of the cells.

### 2.13. Motility Assays

Motility assays were performed as outlined elsewhere [34]. Briefly, *C. jejuni* were grown to mid-log phase overnight in MH broth, pelleted by centrifugation, and suspended in MH broth to an OD_540_ = 1.0. Five microliters of each culture were spotted onto a MH plate containing 0.4% agar, and the plate was incubated for 48 h in a microaerobic chamber at 37 °C. The isolate was judged to be motile if the bacterial growth (migration) was visible beyond the edge of the inoculation spot zone. The plates were imaged with an ImageQuant LAS-4000 mini, and the zone of motility was measured using the ImageJ software. The isolates were tested for motility on six different days. Regardless of the day, the results were consistent.

### 2.14. Statistical Analysis

All statistical analysis was performed with GraphPad Prism 6.0g (La Jolla, CA, USA). The specific statistical tests are indicated in the figure legends.

## 3. Results

### 3.1. Introduction of a Wild-Type Copy of the cadF Gene into the C. jejuni Chromosome

**Figure 1:**
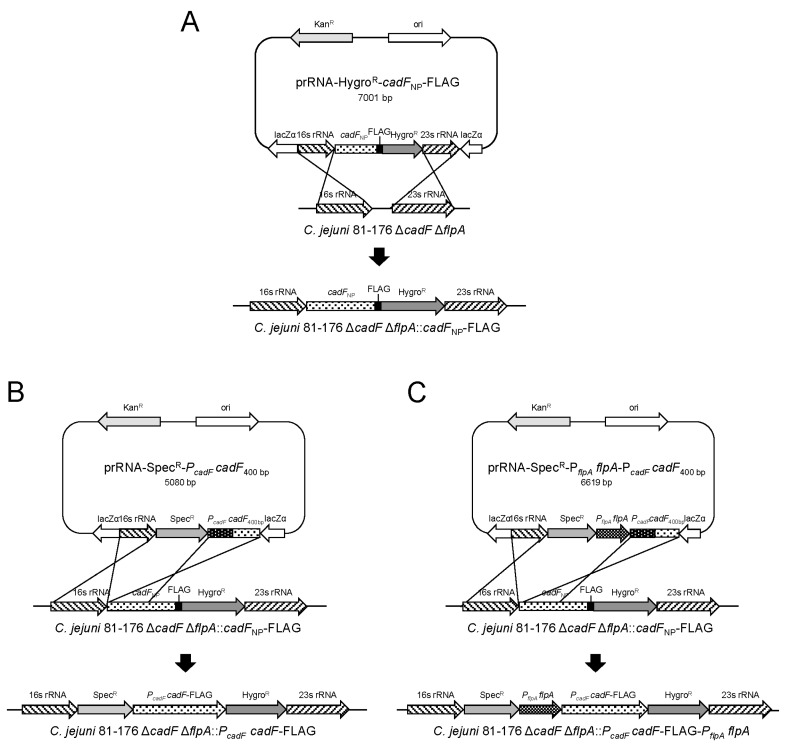
Two-step suicide vector strategy for the introduction of a wild-type copy of the *cadF* and *flpA* genes into the *C. jejuni* chromosome. (**A**) In the first step, a promoterless *cadF* gene fused to a FLAG-tag was cloned into the prRNA-Hygro^R^ vector, resulting in the prRNA-Hygro^R^-*cadF*_NP_-FLAG construct. This construct was introduced into a *C. jejuni* Δ*cadF* mutant or Δ*cadF* Δ*flpA* double mutant by electroporation to generate the isolates that have promoterless *cadF* gene inserted between the 16S rRNA and 23S rRNA genes in the chromosome. (**B**) The second step includes the cloning of DNA fragments containing the *cadF* promoter (*P_cadF_*) and a portion of *cadF* coding sequence from the 5′-end (283 bp, 334 bp, or 400 bp) into a prRNA vector with the 16S rRNA gene and the spectinomycin resistance cassette. Here, the representative image shows the insertion of the c*adF* promoter and the 400 bp of *cadF* 5′-end into the *C. jejuni* Δ*cadF* Δ*flpA*::*cadF*_NP_-FLAG strain. A *cadF*-complemented isolate was generated by recombination with the 16S rRNA gene and the DNA sequence containing the 5′-end of the *cadF* gene. The Hygro^R^ Spec^R^ transformants were confirmed to contain the entire *cadF* gene with its endogenous promoter inserted between the 16S and 23S rRNA genes by PCR analysis. (**C**) A similar approach was taken to complement a Δ*cadF* Δ*flpA* mutant with wild-type copies of *cadF* and *flpA*. In the first step, the promoterless *cadF* gene was incorporated in the rRNA region of the chromosome of the *C. jejuni* Δ*cadF* Δ*flpA* mutant. In the second step, the entire *flpA* gene with its native promoter, the promoter of *cadF*, and a portion of the *cadF* coding sequence from the 5′-end (234 bp, 334 bp, or 400 bp) were inserted in the rRNA region to generate the *cadF flpA* double complement isolate.

To the best of our knowledge, a *C. jejuni cadF* deletion mutant (Δ*cadF*) has yet to be complemented primarily due to the difficulty in generating a shuttle vector; the expression of the full-length *cadF* gene from its endogenous or a heterologous promoter is toxic to *E. coli*. We utilized a two-step procedure whereby a fragment harboring the promoterless *cadF* gene (*cadF_NP_*-FLAG) was first inserted into the 16S and 23S rRNA gene cluster of *C. jejuni*, followed by the introduction of a second fragment harboring the *cadF* promoter upstream of the promoterless *cadF* gene (Figure 1). First, a *C. jejuni* Δ*cadF* mutant harboring a promoterless *cadF* gene fused with a FLAG-tag inserted into the rRNA gene cluster (*C. jejuni* isolate designation: Δ*cadF*::*cadF_NP_*-FLAG). Then, three additional constructs were generated that contained the *C. jejuni* 16S rRNA gene, the spectinomycin antibiotic resistance gene, and the endogenous *cadF* promoter plus a 5′-end portion of the *cadF* gene. The prRNA-Spec^R^ vector harboring the *cadF* promoter (*P_cadF_*) and 5′-end gene fragments (283 bp, 334 bp, or 400 bp) (*cadF*_283 bp_, *cadF*_334 bp_, or *cadF*_400 bp_) were introduced into the *C. jejuni* by electroporation, and Spec^R^ clones were selected on agar plates. The primary reason for amplifying the different 5′-end gene fragments was in case one or more of the partial gene fragments proved to be toxic when expressed in *E. coli*; nevertheless, all three fragments were successfully cloned into the prRNA-Spec^R^ vector. The *C. jejuni* Hygro^R^ Spec^R^ transformants were confirmed to contain the entire *cadF* gene with its endogenous promoter inserted between the 16S and 23S rRNA genes by PCR analysis (*C. jejuni* isolate designation: Δ*cadF*::*P_cadF_ cadF*-FLAG) (data not shown). A similar approach was taken to introduce the wild-type copy of *cadF* in Δ*cadF* Δ*flpA* double mutant (*C. jejuni* isolate designation: Δ*cadF* Δ*flpA*::*P_cadF_ cadF*-FLAG). The innovative aspect of this approach is that the construct containing the coding sequence of the *cadF* gene did not contain its endogenous promoter, so it was not expressed in *E. coli*.

### 3.2. Complementation of cadF in a C. jejuni cadF Deletion Mutant

**Figure 2:**
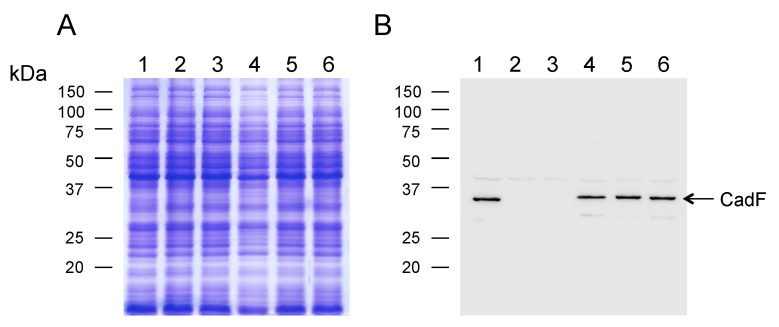
Electrophoretic and immunoblot analysis of *C. jejuni* Δ*cadF* transformants. Bacterial whole-cell lysates were separated in SDS 12.5% polyacrylamide gels and either: (**A**) stained with Coomassie Brilliant Blue R-250 or (**B**) transferred to polyvinylidene fluoride membranes and probed with a rabbit α-CadF serum. The positive control consisted of a *C. jejuni* wild-type strain, and the negative controls consisted of a mutant with a *cadF* gene deletion (Δ*cadF*) and a mutant with a promoterless *cadF* gene fused to a FLAG-tag (Δ*cadF*::*cadF*_NP_-FLAG). The arrow indicates the CadF protein. Lanes: *C. jejuni* strains (1) 81-176 (wild-type); (2) Δ*cadF*; (3) Δ*cadF*::*cadF*_NP_-FLAG; (4) Δ*cadF*::*P_cadF_*_-283 bp_ *cadF*-FLAG; (5) Δ*cadF*::*P_cadF_*_-334 bp_ *cadF*-FLAG; and (6) Δ*cadF*::*P_cadF_*_-400 bp_ *cadF*-FLAG. Molecular mass standards (in kDa) are on the left.

Protein synthesis from the *cadF* gene in the background of the *C. jejuni* Δ*cadF* mutant was examined by SDS-PAGE coupled with immunoblot analysis. A representative gel and immunoblot of the transformants is shown in Figure 2. The positive control consisted of the *C. jejuni* wild-type strain. The negative controls consisted of two Δ*cadF* mutants, one being the Δ*cadF* mutant and the other being the Δ*cadF* mutant harboring the promoterless *cadF* gene (Δ*cadF*::*cadF*_NP_-FLAG). A 37 kDa protein was readily observed in the *C. jejuni* wild-type strain and the three *C. jejuni* Δ*cadF*::*P_cadF_ cadF*-FLAG tagged transformants, as judged by immunoblot analysis using the rabbit α-CadF serum. This finding demonstrates that it is possible to get efficient gene expression and protein synthesis by the integration of a gene with its endogenous promoter within the rRNA gene cluster. Noteworthily, the 37 kDa protein was also detected in the three *C. jejuni* Δ*cadF*::*P_cadF_ cadF*-FLAG transformants by immunoblot analysis using an α-FLAG antibody (not shown), as each contained a FLAG-tag. The addition of an epitope tag provides an additional tool for either the detection or the isolation of a protein.

### 3.3. Complementation of cadF and flpA in a C. jejuni cadF flpA Double Deletion Mutant

**Figure 3:**
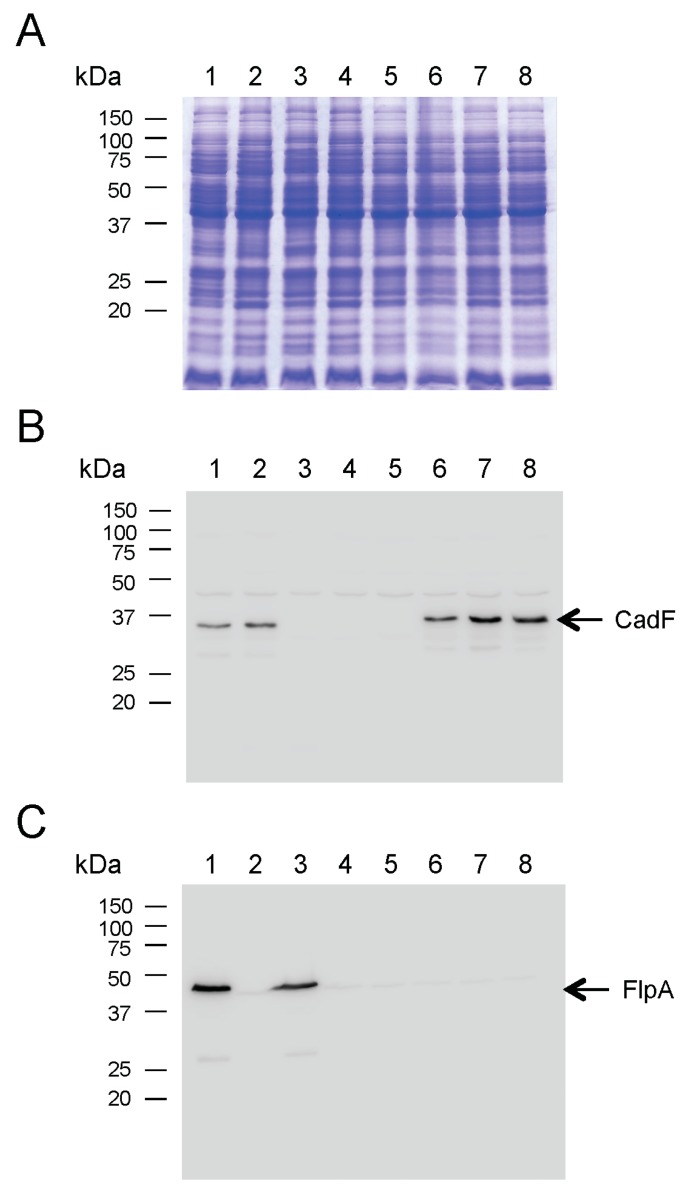
Electrophoretic and immunoblot analysis of *C. jejuni* Δ*cadF* Δ*flpA* transformants. Bacterial whole-cell lysates were separated in SDS 12.5% polyacrylamide gels and either: (**A**) stained with Coomassie Brilliant Blue R-250, (**B**) transferred to polyvinylidene fluoride (PVDF) membranes and probed with a rabbit α-CadF serum, or (**C**) transferred to PVDF membranes and immunoblot with a rabbit α-FlpA serum. The positive control consisted of a *C. jejuni* wild-type strain. The negative controls consisted of a Δ*cadF* Δ*flpA* mutant (lane 4) and the Δ*cadF* Δ*flpA*::*cadF*_NP_-FLAG isolate (lane 5). The arrows indicate the CadF protein (Panel B) and the FlpA protein (Panel C). Lanes: *C. jejuni* strains (1) 81-176 (wild-type); (2) Δ*flpA*; (3) Δ*cadF*; (4) Δ*cadF* Δ*flpA*; (5) Δ*cadF* Δ*flpA*::*cadF*_NP_-FLAG; (6) Δ*cadF* Δ*flpA*::*P_cadF_*_-283 bp_ *cadF*-FLAG; (7) Δ*cadF* Δ*flpA*::*P_cadF_*_-334 bp_ *cadF*-FLAG; and (8) Δ*cadF* Δ*flpA*::*P_cadF_*_-400 bp_ *cadF*-FLAG. Molecular mass standards (in kDa) are on the left.

The ability to restore *cadF* gene expression in a *C. jejuni* Δ*cadF* mutant prompted additional experiments using an identical approach in the *C. jejuni* *cadF flpA* double deletion mutant (Δ*cadF* Δ*flpA*). A representative gel and immunoblot of the transformants is shown in Figure 3. The *C. jejuni* wild-type strain was included as a positive control. The *C. jejuni* Δ*cadF*, Δ*flpA*, and Δ*cadF* Δ*flpA* mutants were included as negative controls. A 37 kDa immunoreactive band was detected with the rabbit α-CadF serum in the *C. jejuni* wild-type strain, Δ*flpA*, and three *C. jejuni* Δ*cadF* Δ*flpA*::*P_cadF_ cadF*-FLAG transformants. In addition, a 46 kDa immunoreactive band was detected with the rabbit α-FlpA serum in only the *C. jejuni* wild-type strain and Δ*cadF* isolate. The immunoblot analysis indicated that the two-step strategy was successful in restoring *cadF* gene expression in the *C. jejuni* Δ*cadF* Δ*flpA* isolate.

### 3.4. CadF and FlpA Localize in the Outer Membrane of the C. jejuni Transformants

**Figure 4:**
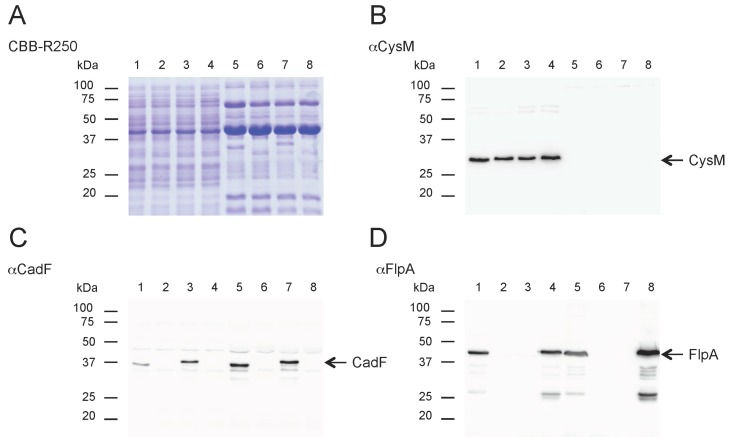
CadF and FlpA are localized in the outer membrane protein (OMP) extracts of the *C. jejuni* transformed isolates. *C. jejuni* whole-cell lysates (WCL) and OMP extracts from *C. jejuni* wild-type, the ∆*cadF* ∆*flpA* isolate, and the ∆*cadF* ∆*flpA* isolate complemented with *cadF* or *flpA* were subjected to SDS-PAGE and immunoblot analysis with rabbit α-CadF, α-FlpA, and α-CysM-specific sera. Panels: (**A**) proteins were stained with Coomassie Brilliant Blue R-250 (CBB-R250) to show equal loading; (**B**) immunoblot probed with the α-CysM serum, a cytosolic protein used to assess the purity of the OMP extracts; (**C**) immunoblot probed with the α-CadF serum; and (**D**) immunoblot probed with the α-FlpA serum. Lanes: *C. jejuni* strains (1) 81-176 (wild-type), WCL; (2) *∆cadF ∆flpA*, WCL; (3) *∆cadF ∆flpA*::*P*_cadF-400 bp_ *cadF*-FLAG, WCL; (4) *∆cadF ∆flpA*::*P*_flpA_ *flpA*, WCL; (5) 81-176 (wild-type), OMP; (6) *∆cadF ∆flpA*, OMP; (7) *∆cadF ∆flpA*::*P*_cadF-400 bp_ *cadF*-FLAG, OMP; (8) *∆cadF ∆flpA*::*P*_flpA_ *flpA*, OMP. The position of the CysM (32 kDa), CadF (37 kDa), and FlpA (46 kDa) proteins are highlighted with arrows. The positions of the molecular mass standards (in kDa) are indicated on the left.

To address the localization of the CadF and FlpA proteins in the *C. jejuni* transformed isolates, *C. jejuni* whole-cell lysates (WCLs) and outer membrane protein (OMP) extracts were prepared from the: (1) 81-176 wild-type strain, (2) Δ*cadF* Δ*flpA* mutant, (3) Δ*cadF* Δ*flpA*::*P_cadF_ cadF*-FLAG isolate, (4) Δ*cadF* Δ*flpA*::*P_flpA_ flpA* isolate; and (5) Δ*cadF* Δ*flpA*::*P_cadF_ cadF*-FLAG-*P_flpA_ flpA* isolate. SDS-PAGE and immunoblot analyses were performed as described in the “Materials and Methods” section. The CadF and FlpA proteins were readily detected in the WCLs and OMP extracts of the appropriate isolates using the rabbit α-CadF and α-FlpA sera (Figure 4). To ensure that the OMP extracts were not contaminated with cytoplasmic proteins, the samples were probed with a rabbit α-*C. jejuni* CysM-specific serum. CysM is a 32.4 kDa cytoplasmic protein involved in cysteine biosynthesis [32]. In contrast to the WCLs, CysM was not detected in the OMP extracts of any of the *C. jejuni* isolates. Collectively, these data indicate that the CadF and FlpA proteins are located in the outer membrane of the *C. jejuni* transformed isolates.

### 3.5. CadF and FlpA are Required for Binding of C. jejuni to Epithelial Cells

**Figure 5:**
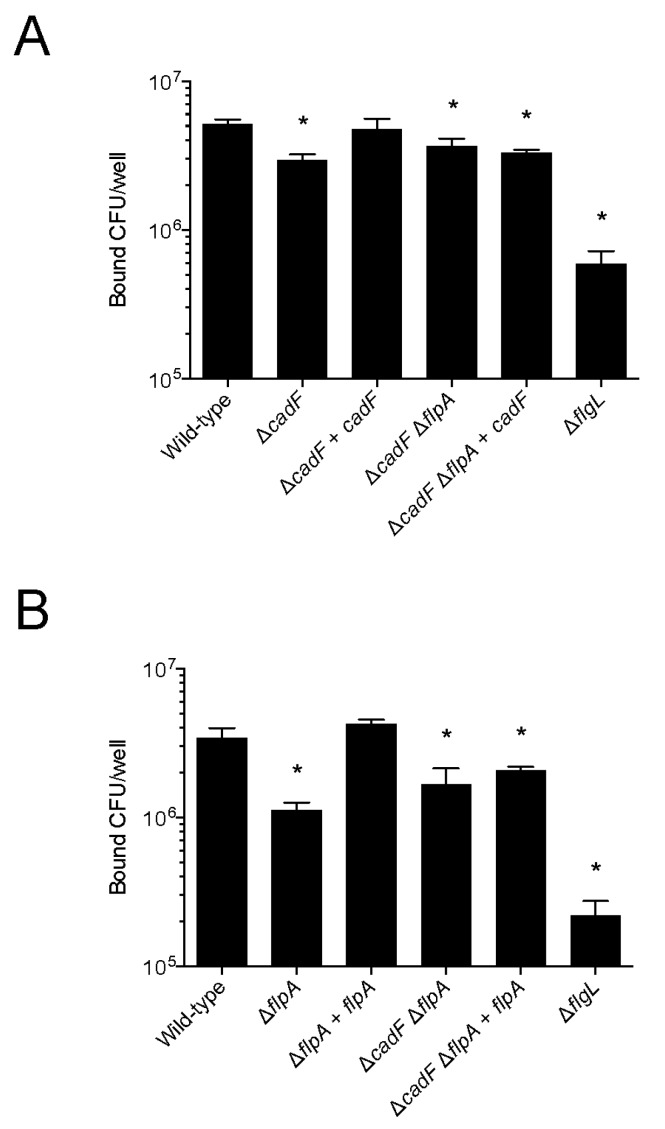
C. *jejuni* adherence to INT 407 cells requires both CadF and FlpA. In vitro adherence assays were performed with human INT 407 epithelial cells and the *C. jejuni* 81-176 wild-type strain, *C. jejuni* Δ*cadF* and Δ*flpA* mutants, and complemented strains as outlined in the “Materials and Methods” section. (**A**) Transformation of a *C. jejuni* Δ*cadF* mutant (Δ*cadF*::*cadF*_NP_-FLAG) but not the Δ*cadF* Δ*flpA* mutant restores binding to the level observed with the wild-type strain. (**B**) Transformation of a *C. jejuni flpA* deletion mutant (Δ*flpA*) but not the Δ*cadF* Δ*flpA* mutant restores binding to the level observed with the wild-type strain. Values represent the number of adherent bacteria ± standard deviation. The asterisks indicate that the number of bacteria bound to the INT 407 cells was statistically different (* *p* < 0.05) from that of the *C. jejuni* wild-type strain, as judged by one-way ANOVA followed by Dunnett’s multiple comparisons test.

The goal of this study was to determine the individual contribution of the CadF and FlpA proteins in *C. jejuni*–host cell interactions. Previous work has revealed that the deletion of either the *cadF* gene or the *flpA* gene results in the reduction of *C. jejuni* binding to host epithelial cells [18,30]. In this study, we wanted to know whether the incorporation of a wild-type copy of either a *cadF* or *flpA* could restore the defect in host cell binding of the *C. jejuni* Δ*cadF* Δ*flpA* double mutant. This question was experimentally addressed by performing a binding assay with INT 407 cells and the *C. jejuni* wild-type strain, Δ*cadF* mutant, Δ*flpA* mutant, Δ*cadF* Δ*flpA* mutant, complemented *cadF* isolate (Δ*cadF* Δ*flpA*::*P_cadF_ cadF*-FLAG), and complemented *flpA* isolate (Δ*cadF* Δ*flpA*::*P_flpA_ flpA*) (Figure 5). As expected, the Δ*cadF* mutant harboring a wild-type copy of the *cadF* gene restored the level of cell binding to that observed with the wild-type strain (Figure 5A). Similarly, the Δ*flpA* mutant harboring a wild-type copy of the *flpA* gene also restored the defect of cell binding (Figure 5B). However, neither *cadF* nor *flpA* rescued the binding defect of the Δ*cadF* Δ*flpA* mutant to that observed with the wild-type strain (Figure 5A,B). This suggests both CadF and FlpA are required for the binding of *C. jejuni* to the host cells.

### 3.6. CadF and FlpA are Cooperative in the Binding to Soluble FN and Epithelial Cells

**Figure 6:**
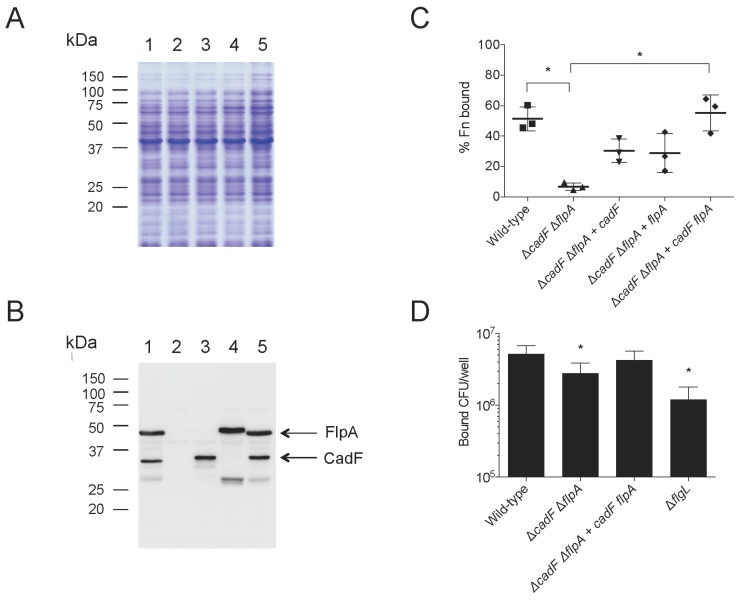
Maximal *C. jejuni* binding to soluble fibronectin (FN) and epithelial cells requires both CadF and FlpA. Bacterial whole-cell lysates were separated in SDS 12.5% polyacrylamide gels and either: (**A**) stained with Coomassie Brilliant Blue R-250 or (**B**) transferred to polyvinylidene fluoride membranes and probed with a mixture of the rabbit α-CadF serum and rabbit α-FlpA serum. The positive control consisted of a *C. jejuni* wild-type strain. Lanes: *C. jejuni* strains (1) 81-176 (wild-type); (2) *∆cadF ∆flpA*; (3) *∆cadF ∆flpA*::*P_cadF_ cadF*; (4) *∆cadF ∆flpA*::*P_flpA_ flp*: and (5) *∆cadF ∆flpA*::*P_cadF_ cadF*-*P_flpA_ flpA.* (**C**) *C. jejuni* binding to soluble FN. *C. jejuni* were incubated with biotin-labeled FN for one hour, pelleted, and the amount of FN that remained in the solution was assayed. The concentration of biotin-labeled FN was determined using a Pierce biotin quantitation kit, as described in the “Materials and Methods” section. Each dot on the chart represents data from independent assays on independent days. The scale is set such that 100% represents complete FN pulldown (measured by assaying PBS only with no FN), and 0% represents no pulldown (measured by assaying FN only, in the absence of *C. jejuni*). The asterisk indicates that the amount of FN-binding was significantly different than the *∆cadF ∆flpA* mutant as tested by one-way ANOVA followed by a post-hoc Dunnett’s analysis (* *p* < 0.05). (**D**) *C. jejuni* binding to epithelial cells. In vitro adherence assays were performed with human INT 407 epithelial cells and the *C. jejuni* 81-176 wild-type strain, Δ*cadF* Δ*flpA* mutant, Δ*cadF* Δ*flpA* mutant complemented with both *cadF* and *flpA*, and the Δ*flgL* mutant. Transformation of a *C. jejuni* Δ*cadF* Δ*flpA* mutant with both *cadF* and *flpA* restores binding to the level observed with the wild-type strain. Values represent the number of adherent bacteria ± standard deviation. The asterisks indicate that the number of bacteria bound to the INT 407 cells was statistically different (* *p* < 0.05) from that of the *C. jejuni* wild-type strain, as judged by one-way ANOVA followed by Dunnett’s multiple comparisons test.

To determine the contribution of CadF and FlpA in *C. jejuni* binding to FN, it was necessary to introduce a wild-type copy of both the *cadF* and *flpA* genes into the Δ*cadF* Δ*flpA* double mutant. The strategy that proved to be successful was the introduction of the *flpA* gene, upstream of the *cadF* gene, into the rRNA cluster using the prRNA-Spec^R^-*P_flpA_* *flpA*-*P_cadF_ cadF*_400 bp_ suicide vector (Figure 1). The resultant colonies were Tet^R^, Kan^R^, Cm^R^, Hygro^R^, and Spec^R^. A representative gel and immunoblot of the transformants are shown in Figure 6, Panels A and B. Importantly, a 46 kDa immunoreactive band was detected with the α-FlpA serum in the *C. jejuni* wild-type strain, the Δ*cadF* Δ*flpA* mutant complemented with the *flpA* genes (*∆cadF ∆flpA*::*P_flpA_ flpA*), and the Δ*cadF* Δ*flpA* mutant complemented with the *cadF* and *flpA* gene (Δ*cadF* Δ*flpA*::*P_cadF_ cadF*-FLAG-*P_flpA_ flpA* isolate). The positive control consisted of the *C. jejuni* wild-type strain, and the negative control consisted of the *C. jejuni* Δ*cadF* Δ*flpA* mutant. Based on the immunoblot analysis, we now possessed three *C. jejuni* isolates: (1) the Δ*cadF* Δ*flpA* mutant containing the *cadF* gene (Δ*cadF flpA*::*P_cadF_ cadF*); (2) the Δ*cadF* Δ*flpA* mutant containing the *flpA* gene (Δ*cadF flpA*::*P_flpA_ flpA*); and (3) the Δ*cadF* Δ*flpA* mutant containing both the *cadF* and *flpA* genes (Δ*cadF* Δ*flpA*:: *P_cadF_ cadF*-*P_flpA_ flpA*).

To determine whether CadF, FlpA, or both CadF and FlpA are required for *C. jejuni* to bind FN at a maximum level (i.e., a level equivalent to that of a wild-type isolate), we compared the FN-binding activity of the Δ*cadF* Δ*flpA* isolate, Δ*cadF* Δ*flpA*::*P_cadF_ cadF* isolate, Δ*cadF* Δ*flpA*::*P_flpA_ flpA* isolate, and the Δ*cadF* Δ*flpA*:: *P_cadF_ cadF*-*P_flpA_ flpA* isolate to a wild-type isolate (Figure 6C). The *C. jejuni* isolates were incubated with biotin-labeled FN for one hour to allow the soluble FN to bind to *C. jejuni*. Quantification of unbound biotin-labeled FN that remained in the solution revealed that neither a *C. jejuni* Δ*cadF* Δ*flpA*::*P_cadF_ cadF* nor a *C. jejuni* Δ*cadF* Δ*flpA*::*P_flpA_ flpA* isolate was able to bind FN to the same extent as observed with the wild-type strain. However, the binding of the *C. jejuni* Δ*cadF* Δ*flpA*::*P_cadF_ cadF*-*P_flpA_ flpA* isolate to FN was indistinguishable from that of the wild-type strain. Based on this finding, we performed a binding assay with INT 407 epithelial cells and compared the binding activity of the Δ*cadF* Δ*flpA* isolate, and the Δ*cadF* Δ*flpA*:: *P_cadF_ cadF*-*P_flpA_ flpA* isolate to a wild-type isolate (Figure 6D). Similar to FN-binding, the *C. jejuni* Δ*cadF* Δ*flpA*::*P_cadF_ cadF*-*P_flpA_ flpA* isolate restored the level of cell binding to that we observed with the wild-type strain. In summary, the FN-binding assay clearly demonstrates that both CadF and FlpA are required for maximal *C. jejuni* binding to FN and epithelial cells.

### 3.7. CadF and FlpA Bind to the 40–45 kDa Fragment of FN

**Figure 7:**
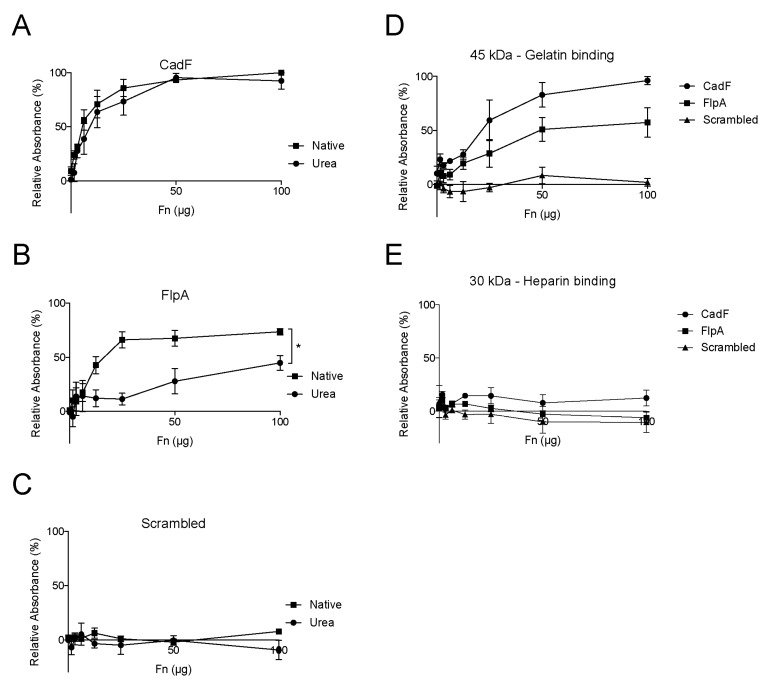
Peptides derived from the binding domains of CadF and FlpA bind to folded fibronectin (FN), specifically in the 45 kDa gelatin-binding region. ELISAs were done using peptides derived from the FN-binding domains of CadF, FlpA, and a scrambled peptide to determine the ability to bind to FN in the presence or absence of 4 M urea. Wells were coated with the peptide, and a range of FN concentrations were added to the wells. (**A**) The CadF peptide binds to FN in a saturation-dependent manner in both the presence and absence of urea. (**B**) The FlpA peptide only binds FN in a saturation-dependent manner in the absence of urea. (**C**) The scrambled peptide, which is a randomized stretch of amino acids drawn from the CadF and FlpA peptides, did not bind to FN. (**D**) Neither the CadF, FlpA, nor scrambled peptide bound to the 30 kDa fragment of FN. (**E**) Both the CadF and FlpA peptides, but not the scrambled peptide, bound to the 45 kDa fragment of FN. The data are presented as a percentage of relative absorbance, where zero is the assay background derived from bovine serum albumin (BSA)-coated wells, and 100% is the highest value in the given assay. The data represents the mean ± the standard deviation of three independent biological replicates. A summary statistic was calculated from the area under the curve (AUC) in the presence and absence of urea and compared using a paired Student’s *t*-test. The asterisk indicates that the AUC was significantly different in the presence and absence of urea (* *p <* 0.05).

FN is a complex glycoprotein composed of multiple repeats (Supplemental Figure S1). Soluble FN has a compact, folded quaternary structure, stabilized through ionic interactions [35]. However, the quaternary structure of FN can be perturbed in response to binding events, including the binding of FN to the surface of cells for fibril assembly and in response to binding by bacterial adhesins [36]. In the laboratory, mild denaturants can destabilize the interionic interactions, leading to an extended (unfolded) conformation [35]. ELISAs were performed to determine if CadF and FlpA displayed different binding properties to folded versus unfolded FN (Figure 7A–C). Wells were coated with peptides derived from the CadF or FlpA binding domains, and a range of FN concentrations were added to the wells in the presence or absence of 4 M urea. The CadF peptide binds to FN in a saturation-dependent manner in both the presence and absence of urea. In contrast, the FlpA peptide bound to FN in a saturation-dependent manner only in the absence of urea. The simplest explanation for this finding is that the optimal binding of FlpA requires FN to be in a folded, or more native, conformational state.

Previous work has demonstrated that FNBPs from Gram-positive and Gram-negative pathogens target different regions of the FN molecule. Indeed, many pathogens target the N-terminal 70 kDa fragment that can be obtained by cathepsin D digestion of FN. Tryptic digestion of the 70 kDa fragment yields the 29–30 kDa N-terminal domain (NTD) harboring the heparin-binding domain and the 40–45 kDa NTD harboring the gelatin-binding domain (Supplemental Figure S1). Previous work has revealed that FlpA binds to the 40–45 kDa fragment. To determine if CadF binds N-terminus of FN, ELISAs were performed using the FlpA peptide as a positive control (Figure 7D,E). Similar to FlpA, CadF exhibited dose-dependent and saturable binding to wells coated with the 45 kDa NTD and a low level of binding (nonspecific) to wells coated with the 30 kDa NTD. Collectively, these data indicate that both CadF and FlpA bind to the 45 kDa NTD but, based on the saturation curves generated in the presence and absence of denaturant (urea), they likely bind to different regions within the FN 45 kDa NTD fragment. Noteworthily, the 40–45 kDa NTD is comprised of four FN I and two FN II repeats (FN I_6_–FN II_1–2_–FN I_7–9_), where FN II_2_ forms a tight complex with FN I_6_ [37,38,39]. Interestingly, the 40–45 kDa NTD has been proposed to undergo major conformational rearrangements [36].

### 3.8. CadF and FlpA Binding to Host-Associated Cellular FN Triggers Host Cell Activation of Erk 1/2

**Figure 8:**
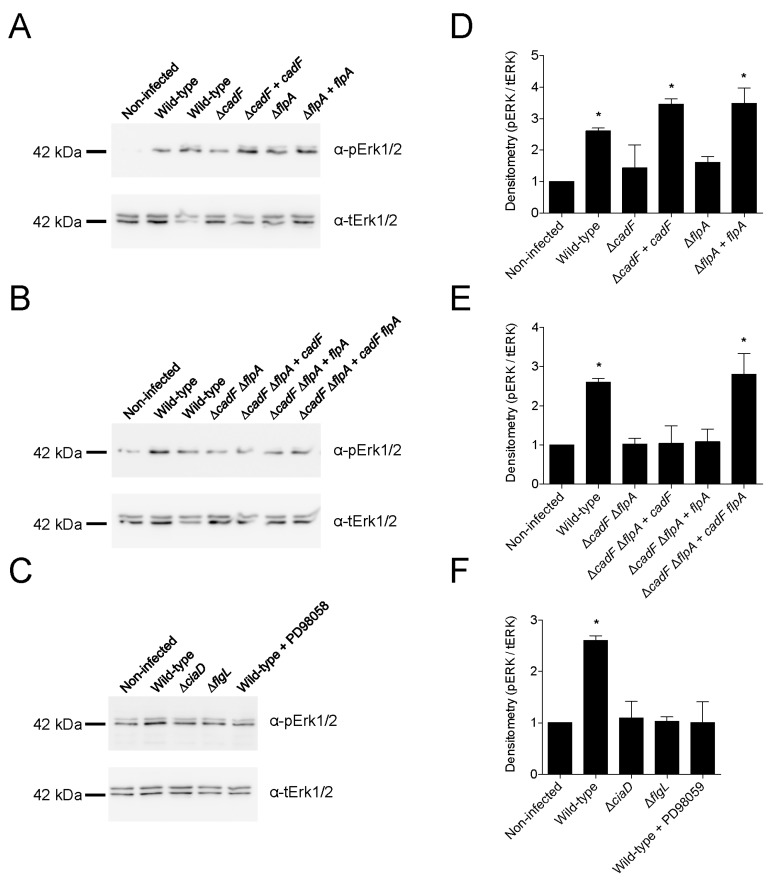
Maximal activation of Erk 1/2 requires the *C. jejuni* CadF and FlpA proteins. INT 407 cells were infected with *C. jejuni* and whole-cell lysates harvested after 60 min incubation as outlined in the “Materials and Methods” section (Panels **A**, **B**, and **C**). Immunoblots were probed with a phospho-specific antibody to Erk 1/2 (*M*_r_ = 42 and 44 kDa). Blots were stripped and re-probed with a total α-Erk 1/2 antibody for a loading control. (Panels **D**, **E**, and **F**) Densitometry was performed on the immunoblots to determine the level of Erk 1/2 activation. The value obtained for INT 407 cells, in the absence of *C. jejuni* infection, was established as a value of one. Please note two wells were infected with the *C. jejuni* wild-type isolate in the top two blots (Panels **A** and **B**). The asterisks indicate a significant difference in values compared to the control (INT 407 cells only), as judged by one-way ANOVA followed by a post-hoc Dunnett’s analysis (* *p* < 0.05). Error bars represent standard deviations of samples from three biological replicates.

*C. jejuni* internalization into host cells is a bacterial-driven process that requires activation of the mitogen-activated protein kinase (MAPK) pathway. More specifically, activated (phosphorylated) Erk 1/2 activates cortactin, which, in turn, binds to the actin remodeling proteins N-WASP and WAVE2 to promote membrane ruffling [40]. To address if the CadF and FlpA proteins are necessary for the activation of the host cell kinase Erk 1/2, INT 407 cells were infected with a *C. jejuni* wild-type strain (positive control), the *cadF* and *flpA* mutants and complemented isolates, and a Δ*flgL* mutant (negative control). The *flgL* gene encodes the hook-filament junction protein, and the FlgL protein is required for both bacterial motility and *Campylobacter* effector protein (Cia protein) export from the flagellar T3SS [33]. The results of the assays are shown in Figure 8. The infection of INT 407 cells with the *C. jejuni* wild-type strain resulted in a significant increase in phosphorylated Erk 1/2 when compared to uninfected cells. In contrast to the wild-type isolate, phosphorylated Erk 1/2 was not increased in cells infected with the Δ*cadF* mutant, Δ*flpA* mutant, Δ*cadF* Δ*flpA* mutant, nor the Δ*cadF* Δ*flpA* mutant harboring a wild-type copy of the *cadF* or *flpA* gene (Figure 8A,B,D,E). The necessity of CadF and FlpA for Erk 1/2 activation was demonstrated using complemented isolates (Figure 8, Panels A, B, D, and E). Previous work has demonstrated that the *C. jejuni* CiaD effector protein, which is secreted from the flagellar T3SS, is required for maximal cell invasion via Erk 1/2 activation and for *C. jejuni* disease in a mouse model [40,41]. As expected, Erk 1/2 activation was blunted in the INT 407 cells inoculated with the *C. jejuni ciaD* deletion mutant (Δ*ciaD*) and *flgL* deletion mutant (Δ*flgL*) (Figure 8C,F). Finally, the inoculation of the INT 407 cells with the *C. jejuni* wild-type strain in the presence of the MEK 1/2 inhibitor PD98059 (a potent inhibitor of Erk 1/2 activation) prevented Erk 1/2 activation (Figure 8C,F). These results demonstrate that the *C. jejuni* CadF and FlpA proteins are both necessary for maximal Erk 1/2 activation.

### 3.9. C. jejuni Effector Protein Delivery to Host Cells Requires CadF and FlpA

**Figure 9:**
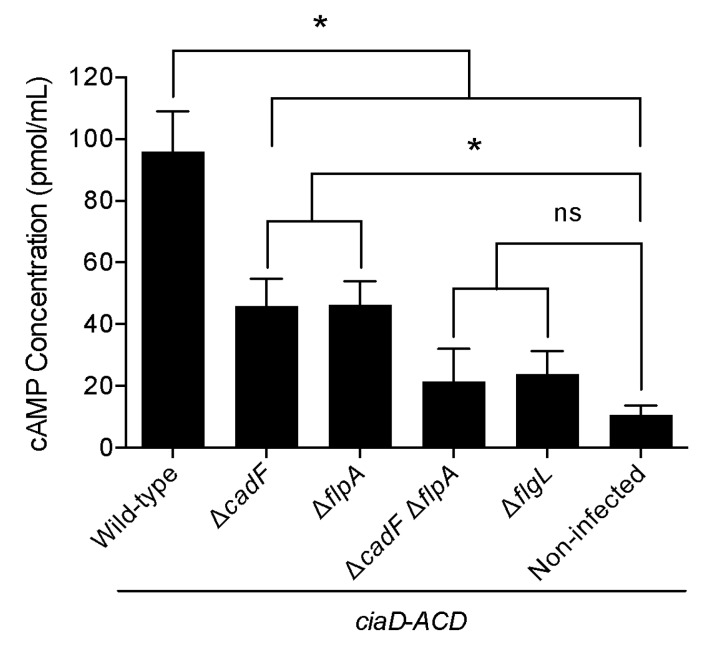
Efficient delivery of the *C. jejuni* CiaD effector protein to host cells requires the CadF and FlpA adhesins. A *C. jejuni* wild-type strain, Δ*cadF* mutant, Δ*flpA* mutant, Δ*cadF* Δ*flpA* mutant, and Δ*flgL* mutant (negative control) were transformed with shuttle vector harboring *ciaD* fused to the adenylate cyclase domain (*ciaD*-*ACD*). The delivery of the CiaD-ACD fusion proteins to the cytosol of human INT 407 cells was determined using the adenylate cyclase domain (ACD) delivery assay, as described in the “Materials and Methods” section. The asterisk indicates that the amount of cAMP produced in cells infected with the *C. jejuni* wild-type strain was significantly greater than the value obtained from a given mutant, as judged by one-way ANOVA followed by Tukey’s analysis (* *p*< 0.05). The amount of cAMP produced in cells infected with the *C. jejuni* Δ*cadF* mutant and Δ*flpA* mutant was also significantly greater than the non-infected samples (* *p*< 0.05). The values obtained from the *C. jejuni* Δ*cadF* Δ*flpA* mutant, and Δ*flgL* mutant showed no statistical significance compared to non-infected samples as judged by one-way ANOVA followed by Tukey’s analysis (* *p*< 0.05; ns, non-significant). The data represent the mean ± standard deviation of the values from three biological replicates.

*C. jejuni* synthesizes at least eight flagellar-dependent secreted virulence proteins, known as the *Campylobacter* invasion antigens (Cia), that are delivered to the cytosol of host cells where they modify signaling processes [42,43]. We speculated that the MAPK pathway (Erk 1/2) was not activated in INT 407 cells infected with the Δ*cadF* mutant, Δ*flpA* mutant, and Δ*cadF* Δ*flpA* mutant because, in the absence of effective binding, *C. jejuni* cannot deliver effector proteins into the cells. To determine if Cia protein delivery to host cells requires bacteria–host cell contact mediated by the CadF and/or FlpA FNBPs, we used isolates transformed with the *ciaD*-*ACD* construct coupled with the ACD reporter assay. We specifically chose to use a CiaD-ACD fusion protein for these assays because CiaD is the most extensively characterized Cia protein and, as mentioned previously, CiaD is required for maximal Erk 1/2 activation [40,41]. A shuttle vector harboring the *ciaD*-*ACD* construct was introduced into the *C. jejuni* wild-type strain and mutants by conjugation. All *C. jejuni* transformants were confirmed to produce the CiaD-ACD fusion protein (*C. jejuni* wild-type strain (positive control), Δ*cadF* mutant, Δ*flpA* mutant, Δ*cadF* Δ*flpA* mutant, and Δ*flgL* mutant (secretion-negative, negative control)) (Supplemental Figure S2). In addition, all *C. jejuni* transformants, except for the Δ*flgL* mutant, were motile (Supplemental Figure S3). The ability of the *C. jejuni* transformants to deliver the CiaD-ACD protein to INT 407 cells was determined after a 45 min infection. As expected, infection with the *C. jejuni* wild-type strain harboring *ciaD*-*ACD* resulted in a significant increase in cAMP compared to the Δ*flgL* secretion-negative mutant (Figure 9). Regarding the *C. jejuni* FN-binding mutants, infection of the cells with the wild-type strain resulted in a significant increase in cAMP compared to the Δ*cadF*, Δ*flpA*, and Δ*cadF* Δ*flpA* mutants. Notably, infection of the cells with the Δ*cadF* mutant as well as the Δ*flpA* mutant resulted in a moderate increase in cAMP when compared to the Δ*cadF* Δ*flpA* double mutant (Figure 9). Collectively, these results demonstrate that the CiaD effector protein requires the CadF and FlpA proteins to be effectively delivered to the cytosol of host epithelial cells.

## 4. Discussion

**Figure 10:**
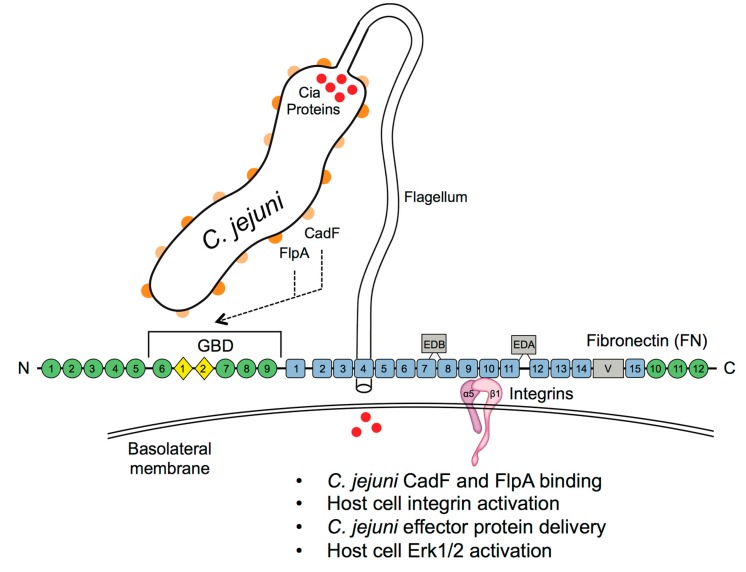
A model of *C. jejuni* interaction with host cells. *C. jejuni* drives the host cell processes by forming the three-component bridge between the CadF and FlpA proteins, host cell-associated fibronectin (FN), and α_5_β_1_ integrin. While only a single FN molecule and a single α_5_β_1_ integrin are shown for simplicity, *C. jejuni-*induced host cell signaling requires multiple FN molecules and the occupancy and clustering of multiple α_5_β_1_ integrin heterodimers for the maximal stimulation of cellular signaling pathways. FN is composed of 12 FN type I repeats (FN I, green circles), 2 FN type II repeats (FN II, yellow diamonds), and 15–18 FN type III repeats (FN III, blue squares). Additionally, Extra Domains A and B (EDA and EDB) are shown and the variable (V) region that connects two type III repeats. Both CadF and FlpA proteins bind to the 45 kDa N-terminal domain (NTD) of FN that harbors the gelatin-binding domain (GBD). However, CadF and FlpA likely bind to different regions of the 45 kDa fragment based on ELISAs performed in the presence or absence of 4 M urea (see the text for details). Intimate contact between *C. jejuni* and the host cells by CadF and FlpA facilitates the delivery of the *C. jejuni* effector protein CiaD, which is necessary for Erk 1/2 activation. Together, CadF and FlpA set the stage for host cell invasion by binding to FN associated with the α_5_β_1_ integrins on the basolateral surface of cells (sites of focal adhesions) and to deliver effectors that stimulate host cellular signaling pathways.

*C. jejuni* synthesizes a repertoire of adhesins (i.e., CadF, FlpA, JlpA, etc.) to promote cell adherence. Among these adhesins, CadF and FlpA are two surface-exposed proteins that bind to the extracellular matrix glycoprotein FN. Previously, both CadF and FlpA were reported to promote *C. jejuni* binding to cultured epithelial cells and were shown to be required for *C. jejuni* to colonize chickens [17,21]. However, the individual role of CadF and FlpA in binding to FN and host cells was not known previous to this study, in part, because researchers had difficulty in generating a CadF complemented isolate. In this study, we utilized a two-step cloning strategy to complement a *C. jejuni* Δ*cadF* mutant. The experiments performed herein revealed that: (1) neither CadF nor FlpA alone could restore the defect in binding of *C. jejuni* Δ*cadF* Δ*flpA* double mutant to host cells; (2) CadF and FlpA binding to FN is additive; (3) both CadF and FlpA are necessary for *C. jejuni* activation of the MAPK signaling pathway (Erk 1/2); and (4) both CadF and FlpA are required for the delivery of the Cia effector proteins into the cytosol of a host cell. A model depicting the *C. jejuni* CadF- and FlpA-driven processes is shown in Figure 10. Noteworthily, both CadF and FlpA proteins are conserved among *C. jejuni* isolates, implying that these two proteins contribute to *C. jejuni* pathogenesis [16,18].

Many bacterial genes and gene fragments have been found to be lethal to *E. coli* when expressed [44]. This introduces a technical limitation when studying these genes and their products. Since the discovery of CadF, researchers have been unable to complement a *C. jejuni cadF* mutant due to the inability to construct a complementation vector. In this study, we developed an innovative approach to complement the *cadF* deletion mutant. Our approach involved generating a suicide vector with a wild-type copy of the *cadF* gene without its promoter in *E. coli*. The promoterless *cadF* gene was then introduced into the rRNA cluster of the *C. jejuni* Δ*cadF* mutant. A second transformation was used to introduce the *cadF* promoter, thereby restoring the expression of the *cadF* gene from its native promoter. The introduction of both fragments into the rRNA region was dependent on homologous recombination using flanking sequences and the use of antibiotic resistance genes to select for the desired recombinants. We propose that the strategy used in this study for complementation can be used for other *C. jejuni* genes that, when expressed in *E. coli*, are toxic. We also believe that this cloning strategy should be broadly applicable to other bacteria.

In accordance with previous studies, we found that a deletion of the *cadF* gene, *flpA* gene, or both genes significantly reduced the number of *C. jejuni* bound to INT 407 cells. Moreover, the CadF and FlpA proteins, which share only 25.6% similarity at the amino acid level, cannot functionally complement one another as evident in the phenotypic assays performed in this study (e.g., facilitating cell adherence, promoting FN binding, stimulating cell signaling, and enabling effector delivery to host cells). This finding is consistent with the fact that the CadF and FlpA proteins have distinct FN-binding domains [19,20]. Noteworthily, deduced amino acid sequences of the *S. aureus* FNBPA and FNBPB share a high level (74.2%) of similarity [45], and that complementation of a *fnbA fnbB* double mutant with either *fnbA* or *fnbB* fully restores the FN-binding activity [46]. Collectively, the data indicate that CadF and FlpA work together to maximize the binding of *C. jejuni* to host-associated FN.

The FN molecule is composed of multiple copies of three repeats or modules, each having a well-organized secondary and tertiary structure [36]. In plasma, FN is present in a compact form where the quaternary structure is facilitated through the interactions of specific modules. However, the binding of FN to cells or, more relevant to this study, the binding of bacterial FNBPs to FN, results in perturbation of the quaternary structure and an extended molecule (unfolded conformation). Moreover, studies have revealed that bacterial FNBPs bind to different sites (domains) within the FN molecule [38]. The 29 kDa N-terminal fragment of FN is the most prominent and canonical FN region targeted by bacterial FNBPs, such as FNBPA and FNBPB in *S. aureus*, and F1 and Sfb1 in *S. pyogenes* [47,48]. The 29 kDa fragment contains five FN I repeats (FN I_1–5_) and harbors the heparin-binding domain. The adjacent 40–45 kDa fragment contains the gelatin-binding domain (GBD)/collagen-binding domain and functions as the non-canonical binding site for select bacterial FNBPs, including the F1 and Sfb1 from *S. pyogenes*, which also bind to the 29 kDa fragment [49]. The 40–45 kDa FN fragment is comprised of four FN I and two FN II repeats in the following order: FN I_6_–FN II_1–2_–FN I_7–9_. Bacterial FNBPs also target the 120–140 kDa central fragment of FN (FN III_9–10_, FN III_12_). For example, FbpA and FbpB from *Clostridium perfringens* bind to FN III_9–10_, and Embp from *Staphylococcus epidermidis* binds to FN III_12_ [50,51]. The differences in the CadF and FlpA FN-binding domains prompted experiments to determine the region that CadF binds to FN. Prior to this study, FlpA was reported to bind to the FN fragment harboring the GBD. Our study revealed that both CadF and FlpA bind to this 45 kDa fragment of FN. However, differences were apparent in the binding properties of the CadF and FlpA peptides. Although the saturation curve for CadF binding to FN was nearly identical in the absence of urea (folded conformation) and presence of urea (unfolded conformation), the binding of the FlpA peptide was noticeably different in the absence versus the presence of urea. These findings suggest that FlpA binds to a conformation-dependent site that is disrupted when FN is unfolded. As mentioned above, FN is folded when secreted into the extracellular space but, upon binding to the α_5_β_1_ integrins, it unfolds. The extended form of FN exposes cryptic binding sites that promote the assembly of FN molecules into fibrils [52]. We speculate that, even though CadF and FlpA both bind to the 45 kDa GBD fragment, they modulate the behavior of FN in different ways. Additional studies are required to determine if CadF and FlpA binding to FN is sequential, or if the binding of one of adhesin can potentiate the binding of the second adhesin.

Bacteria commonly invade (nonphagocytic) epithelial cells via a zipper (exclusive) or a trigger (inclusive) mechanism [53]. The zipper mechanism involves the interaction of bacterial adhesins binding to host cell receptors, resulting in the activation of host cell signaling cascades and bacterial internalization. The trigger mechanism involves the translocation of bacterial effectors into the cytosol of a host cell, which stimulates host cell signaling cascades and massive cytoskeleton rearrangement that drives the formation of membrane ruffles, ultimately leading to bacterial uptake. *S. aureus* uses the FNBPs to facilitate cell internalization by a zipper mechanism [54]. The FNBP-mediated mechanism of *S. aureus* internalization requires FN as a bridging molecule and the α_5_β_1_ integrin as a host cell receptor, resulting in signal transduction, tyrosine kinase activity, and cytoskeletal rearrangements [38,55]. In *S. aureus*, *fnbA* and *fnbB* single mutants show no significant reduction in adhesion to FN [46], but both FNBPs are required for severe disease in mice [56]. Compared with other known and putative *C. jejuni* adhesins, a distinguishing feature of the CadF and FlpA proteins is that they promote cellular invasion by binding to FN bound to α_5_β_1_ integrins on the basolateral surface of a cell [57,58,59]. However, in the early 1990s, it was also reported that *C. jejuni* invasion, but not cell adherence, was significantly reduced in the presence of chloramphenicol, a selective inhibitor of bacterial protein synthesis [60]. Later it was found that *C. jejuni* secretes effector proteins, albeit via its flagellum rather than a dedicated secretory system, and that knockouts in genes encoding the effector proteins blunted *C. jejuni* host cell invasion [25,40,41,42]. Together, these findings indicate that *C. jejuni* utilizes aspects of both the zipper and trigger mechanisms—in essence, a hybrid of the two processes [61].

The finding that both CadF and FlpA are required for maximal binding to FN raised the possibility that both proteins were required for the delivery of the Cia effector proteins into the cytosol of a host target cell. The experiments performed in this study revealed that CadF and FlpA are bi-functional proteins that act in a cooperative manner to first bind to host cells and then to stimulate signal transduction pathways, including the MAPK pathway, involved in *C. jejuni* cell invasion. More precisely, the delivery of the CiaD effector protein to the cytosol of host cells stimulates Erk 1/2 activation, which, in turn, phosphorylates (activates) cortactin [40,41]. Activated cortactin promotes membrane ruffles by binding to the actin remodeling proteins N-WASP and WAVE2 [62]. In support of this model of *C. jejuni* cell invasion, mutagenesis studies have also indicated that CadF and an intact flagellum are involved in Rho GTPase activation and host cell invasion [63,64]. Our findings support a binding and effector mechanism of *C. jejuni* cell invasion, whereby the CadF and FlpA proteins facilitate the delivery of effector proteins.

In a recent study, the contribution of the CadF adhesin to disease was assessed by infecting abiotic (gnotobiotic) IL-10^−/−^ mice with the *C. jejuni* 81-176 wild-type strain and with a *cadF* deletion mutant [65]. In this model, the animals were inoculated with *C. jejuni* (10^9^ CFU on two consecutive days), and disease parameters were assessed daily until six days post-infection. Pathology was scored based on the presence of blood in the stool, diarrhea, and animal behavior. Interestingly, the median pathology score at day six in the mice infected with the Δ*cadF* mutant was approximately eight, while the wild-type infected mice had more severe symptoms with a median pathology score of 12. In addition, there was attenuated intestinal tumor necrosis factor alpha (TNF-α) and interferon gamma (IFN-γ) responses during infection with the Δ*cadF* mutant compared to the wild-type strain. Thus, the Δ*cadF* mutant was blunted in clinical symptoms in mice administered high bacterial doses (i.e., an inoculum that a human is unlikely to encounter). The diminished clinical symptoms observed in the mice inoculated with the Δ*cadF* mutant are consistent with a model whereby the CadF protein contributes to disease by facilitating cell adherence and delivery of the effector proteins. However, given that *C. jejuni* possesses multiple adhesins and that cell adherence is multifactorial, it is possible to bypass the necessity for the intimate cell adherence via CadF, and possibly FlpA, by using high/repeated doses of mutant bacteria. Noteworthily, previous work has also demonstrated that the CadF adhesin, as well as intact flagella, is necessary for Cdc42 and Rac1 activation (GTP-bound) in human INT 407 cells [66,67]. Cdc42 and Rac1 are the primary host cell Rho GTPases involved in *C. jejuni* invasion. We have also reported that Rac1-GTP production is also diminished in human INT 407 cells infected with *C. jejuni flpA* and *cadF flpA* double mutant [19]. Given the findings from this study, which demonstrate that CadF and FlpA work together in facilitating cell adherence, promoting FN binding, stimulating cell signaling, and enabling effector delivery to host cells, studies are warranted to determine if disease symptoms arise in animals inoculated with a *C. jejuni cadF flpA* double mutant.

## 5. Conclusion

In agreement with previous studies, this study further cements the concept that CadF and FlpA are the major FNBPs that promote *C. jejuni* attachment to host cells. With the complementation of *cadF* in Δ*cadF* and Δ*cadF* Δ*flpA* isolates, we demonstrated that *cadF* or *flpA* alone are unable to fully restore the defect in host cell FN-binding. Our data also suggest that CadF and FlpA bind to different sites within the GBD fragment of FN, indicating the cooperative role of these two proteins. Together, the CadF and FlpA adhesins facilitate the binding of *C. jejuni* to host cells, permit the delivery of *C. jejuni* effector proteins into the cytosol of a host target cell, and aid in the rewiring of host cell signaling pathways that alter host cell behavior. *C. jejuni* utilizes the unique binding and effector mechanism, where the coordinated action of the CadF and FlpA FNBPs and Cia effector proteins promote the *C. jejuni* invasion of cells, and modulates host cell signaling. On a final note, we hope that the genetic strategy described herein for gene complementation can be applied to genetic studies with *C. jejuni* and other bacteria with less well-developed genetic systems.

## Figures and Tables

**Table 1:** *C. jejuni* isolates used in this study.

Isolate	Description	Plasmid Used to Generate the Isolate	Antibiotic Resistance ^1^	Reference
81-176	Wild-type strain	None	Tet^R^	[23]
Δ*flgL*	*flgL* deletion mutant	pBSK-Kan2-*flgL*-CAT	Tet^R^ Cm^R^	[24]
Δ*cadF*	*cadF* deletion mutant	pBSK-Kan2-*cadF*-CAT	Tet^R^ Cm^R^	This study
Δ*flpA*	*flpA* deletion mutant	pBSK-*flpA*-Kan2	Tet^R^ Kan^R^	This study
Δ*cadF* Δ*flpA*	*cadF flpA* double deletion mutant	pBSK-*flpA*-Kan2	Tet^R^ Cm^R^ Kan^R^	This study
Δ*cadF*::*cadF*_NP_-FLAG	*cadF* deletion mutant with promoterless *cadF* gene fused to a FLAG-tag	prRNA-Hygro^R^-*cadF*_NP_-FLAG	Tet^R^ Cm^R^ Hygro^R^	This study
Δ*cadF*::*P_cadF_*_-400 bp_ *cadF*-FLAG	*cadF* deletion mutant harboring a wild-type copy of *cadF*	prRNA-Spec^R^-*P_cadF_ cadF*_400 bp_	Tet^R^ Cm^R^ Hygro^R^ Spec^R^	This study
Δ*cadF*::*P_cadF_*_-334 bp_ *cadF*-FLAG	*cadF* deletion mutant harboring a wild-type copy of *cadF*	prRNA-Spec^R^-*P_cadF_ cadF*_334 bp_	Tet^R^ Cm^R^ Hygro^R^ Spec^R^	This study
Δ*cadF*::*P_cadF_*_-283 bp_ *cadF*-FLAG	*cadF* deletion mutant harboring a wild-type copy of *cadF*	prRNA-Spec^R^-*P_cadF_ cadF*_283 bp_	Tet^R^ Cm^R^ Hygro^R^ Spec^R^	This study
Δ*flpA*::*P_flpA_ flpA*	*flpA* deletion mutant harboring a wild-type copy of *flpA*	prRNA-Hygro^R^-*P_flpA_ flpA*	Tet^R^ Kan^R^ Hygro^R^	This study
Δ*cadF* Δ*flpA*::*cadF*_NP_-FLAG	*cadF flpA* double deletion mutant with a promoterless *cadF* gene fused to a FLAG-tag	prRNA-Hygro^R^-*cadF*_NP_-FLAG	Tet^R^ Cm^R^ Kan^R^ Hygro^R^	This study
Δ*cadF* Δ*flpA*::*P_cadF_*_-400 bp_ *cadF*-FLAG	*cadF flpA* double deletion mutant harboring a wild-type copy of *cadF*	prRNA-Spec^R^-*P_cadF_ cadF*_400 bp_	Tet^R^ Cm^R^ Kan^R^ Hygro^R^ Spec^R^	This study
Δ*cadF* Δ*flpA*::*P_cadF_*_-334 bp_ *cadF*-FLAG	*cadF flpA* double deletion mutant harboring a wild-type copy of *cadF*	prRNA-Spec^R^-*P_cadF_ cadF*_334 bp_	Tet^R^ Cm^R^ Kan^R^ Hygro^R^ Spec^R^	This study
Δ*cadF* Δ*flpA*::*P_cadF_*_-283 bp_ *cadF*-FLAG	*cadF flpA* double deletion mutant harboring a wild-type copy of *cadF*	prRNA-Spec^R^-*P_cadF_ cadF*_283 bp_	Tet^R^ Cm^R^ Kan^R^ Hygro^R^ Spec^R^	This study
Δ*cadF* Δ*flpA*::*P_flpA_ flpA*	*cadF flpA* double deletion mutant harboring a wild-type copy of *flpA*	prRNA-Hygro^R^-*P_flpA_ flpA*	Tet^R^ Cm^R^ Kan^R^ Hygro^R^	This study
Δ*cadF* Δ*flpA*::*P_cadF_* *cadF*-FLAG-*P_flpA_ flpA*	*cadF flpA* double deletion mutant harboring a wild-type copies of *cadF* and *flpA*	prRNA-Spec^R^-*P_flpA_* *flpA*-*P_cadF_* *cadF*_400 bp_	Tet^R^ Cm^R^ Kan^R^ Hygro^R^ Spec^R^	This study
81-176 c*iaD*-*ACD*	Wild-type strain harboring *ciaD*-*ACD* fusion vector	pRY111-Hygro^R^-*P_cysM_* *ciaD*-*ACD*	Tet^R^ Hygro^R^	This study
Δ*cadF ciaD*-*ACD*	*cadF* deletion mutant harboring *ciaD*-*ACD* fusion vector	pRY111-Hygro^R^-*P_cysM_* *ciaD*-*ACD*	Tet^R^ Cm^R^ Hygro^R^	This study
Δ*flpA ciaD*-*ACD*	*flpA* deletion mutant harboring *ciaD*-*ACD* fusion vector	pRY111-Hygro^R^-*P_cysM_* *ciaD*-*ACD*	Tet^R^ Kan^R^ Hygro^R^	This study
Δ*cadF* Δ*flpA ciaD*-*ACD*	*cadF flpA* double deletion mutant harboring *ciaD*-*ACD* fusion vector	pRY111-Hygro^R^-*P_cysM_* *ciaD*-*ACD*	Tet^R^ Cm^R^ Kan^R^ Hygro^R^	This study
Δ*flgL ciaD-ACD*	*flgL* deletion mutant harboring *ciaD*-*ACD* fusion vector	pRY111-Hygro^R^-*P_cysM_* *ciaD*-*ACD*	Tet^R^ Cm^R^ Hygro^R^	This study

^1^ Cm^R^: Chloramphenicol; Hygro^R^: Hygromycin; Kan^R^: Kanamycin; Spec^R^: Spectinomycin; Tet^R^: Tetracycline.

**Table 2:** Bacterial plasmids used in this study.

Plasmid	Description	Antibiotic Resistance ^1^	Reference
pBSK-Kan2	pBlueScript II SK (+) cloning vector with the *aphA-3* gene cassette encoding Kan^R^ (Kan2) replacing the original ampicillin resistance cassette	Kan^R^	[25]
pBSK-Kan2-*cadF-*CAT	pBSK-Kan2 suicide plasmid harboring 5′ and 3′ *cadF* flanking fragments with a chloramphenicol resistance cassette (CAT)	Cm^R^ Kan^R^	This study
pBSK-*flpA*-Kan2	pBSK suicide plasmid harboring 5′ and 3′ *flpA* flanking fragments with the *C. jejuni* kanamycin resistance gene (*aphA-3*, Kan2)	Amp^R^ Kan^R^	This study
prRNA-Hygro^R^	pBSK-Kan2 vector harboring the 16S rRNA and the 23S rRNA gene fragments with a hygromycin B resistance cassette	Kan^R^ Hygro^R^	[26]
prRNA-Hygro^R^-*cadF*_NP_-FLAG	prRNA-Hygro^R^ suicide vector harboring the promoterless *cadF* gene fused with a FLAG-tag	Kan^R^ Hygro^R^	This study
prRNA-Spec^R^-*P_cadF_ cadF*_400 bp_	prRNA-Spec^R^ suicide vector harboring 400 bp of the *cadF* gene at the 5′ end with its native promoter	Kan^R^ Spec^R^	This study
prRNA-Spec^R^-*P_cadF_ cadF*_334 bp_	prRNA-Spec^R^ suicide vector harboring 334 bp of the *cadF* gene at the 5′ end with its native promoter	Kan^R^ Spec^R^	This study
prRNA-Spec^R^-*P_cadF_ cadF*_283 bp_	prRNA-Spec^R^ suicide vector harboring 283 bp of the *cadF* gene at the 5′ end with its native promoter	Kan^R^ Spec^R^	This study
prRNA-Hygro^R^-*P_flpA_ flpA*	prRNA- Hygro^R^ suicide vector harboring the *flpA* gene with its native promoter	Kan^R^ Hygro^R^	This study
prRNA-Spec^R^-*P_flpA_* *flpA*-*P_cadF_ cadF*_400 bp_	prRNA-Spec^R^ suicide vector harboring the *flpA* gene and 400 bp of the *cadF* gene at the 5′ end with their native promoters	Kan^R^ Spec^R^	This study
pRY111-Hygro^R^-*P_cysM_* *ciaD*-*ACD*	pRY111-Hygro^R^ shuttle vector harboring the *ciaD* gene with a *C. jejuni cysM* promoter and fused with the adenylate cyclase domain (ACD) coding sequence	Hygro^R^	This study

^1^ Amp^R^: Ampicillin; Cm^R^: Chloramphenicol; Hygro^R^: Hygromycin; Kan^R^: Kanamycin; Spec^R^: Spectinomycin.

**Table 3:** Primers used in this study.

Primer ID	Oligo Name	Sequence 5′-3′	Purpose
MEK4063	81-176-CadF-Dwn-RV	TAT AGG GCG AAT TGG GTA CCG CAG CCT CAT TTC CGT CC	*cadF* mutant construction
MEK4064	81-176-CadF-Dwn-FW	GAT CGG ATC CCC TCG CTC AAG CAA TGA CAC	*cadF* mutant construction
MEK4065	81-176-CadF-CAT-RV	TTG AGC GAG GGG ATC CGA TCT GCG CCC TTT AGT	*cadF* mutant construction
MEK4066	81-176-CadF-CAT-FW	TTG GCA AGT GGC TAG CGT GTT CCT TTC CAA GTT AAT TGC G	*cadF* mutant construction
MEK4067	81-176-CadF-Up-RV	ACA CGC TAG CCA CTT GCC AAA CCT AAA CAT AAT A	*cadF* mutant construction
MEK4068	81-176-CadF-Up-FW	GGG AAC AAA AGC TGG AGC TCC AGT TAG AGG TAT GCT TCC TA	*cadF* mutant construction
MEK4069	81-176-FlpA-Dwn-FW	TAT AGG GCG AAT TGG GTA CCT CTG CTC TAT TTT TTT CAA ATC C	*flpA* mutant construction
MEK4070	81-176-FlpA-Dwn-RV	GCT TGG ATC CAG AAC CTT CAA GCA AAG TTA AGG	*flpA* mutant construction
MEK4071	81-176-FlpA-Kan-FW	TGA AGG TTC TGG ATC CAA GCT TTT TAG ACA TCT AAA TCT AGG TAC TA	*flpA* mutant construction
MEK4072	81-176-FlpA-Kan-RV	AAA GAT TTC GGC TAG CGA TAA ACC CAG CGA ACC	*flpA* mutant construction
MEK4073	81-176-FlpA-Up-FW	TAT CGC TAG CCG AAA TCT TTT CAT CAT TCT CTC C	*flpA* mutant construction
MEK4074	81-176-FlpA-Up-RV	GGG AAC AAA AGC TGG AGC TCA ACT TTT TTA GTA GAT GAA AAT TCA AGG	*flpA* mutant construction
MEK4507	noProm-CadF-XbaI-FW	ATA TTC TAG AAT GAA AAA AAT ATT ATT ATG TTT AGG TTT GGC AAG TGT TTT ATT CAG TGC	*cadF* complement first construct
MEK4508	CadF-nostop-BamHI-RV	ATA TAT GGA TCC TCT TAA AAT AAA TTT AGC ATC CAC TCT TCT ATT ATC CGC TCT ACC TTC	*cadF* complement first construct
MEK4509	rRNA-up-SpecR-XbaI-FW	TGG ATC ACC TCC TTT CTA GAG CTG TTT TTT ACT TGA TAT TGT TTT TTA AAT ATG CTA AAA TTA GGC GTT TC	*cadF* complement second construct
MEK4518	pCadF-SpecR-SacII-RV	GCT TCT TCC CGC GGT TGC TAC TCT GTT CTA AGT AAT TCC TCA ATT TGT TTT TTC	*cadF* complement second construct
MEK4541	pCadF-SacII-FW	GAG TAG CAA CCG CGG GAA GAA GCC CAC AAT TCT CTA AAC G	*cadF* complement second construct
MEK4542	CadF-400bp-SacI-RV	GAA CAA AAG CTG GAG CTC ATT TTA CAC CCG CGC CAT AAT GTC C	*cadF* complement second construct
MEK4543	CadF-334bp-SacI-RV	GAA CAA AAG CTG GAG CTC CCT CAT ATC CTC CAC CTG CTA AAC C	*cadF* complement second construct
MEK4544	CadF-283bp-SacI-RV	GAA CAA AAG CTG GAG CTC CAA TAC CTT TAA TAG CAC TCA AAT AAG TTC TTG TAA TAT C	*cadF* complement second construct
MEK4602	FlpA-Comp-XbaI-FW	GTA TTC TAG AAC AGG AAG AAC TCA TCA AAT TAG AGC	*flpA* complement first construct
MEK4603	FlpA-Comp-BamHI-RV	ACT CGG ATC CCC AAA GAA ATT CAA CAT CAT CCT TGC	*flpA* complement first construct
MEK4676	FlpA-Comp-SacII-FW	GTA TCC GCG GAC AGG AAG AAC TCA TCA AAT TAG AGC	*flpA* complement second construct
MEK4677	FlpA-Comp-SacII-RV	ACT CCC GCG GCC AAA GAA ATT CAA CAT CAT CCT TGC	*flpA* complement second construct
MEK4721	pRY111-HygR-NcoI-FW	ATA GAA GAT CTC CAT GGA CTA AAG CTC TTG CCC AAG AAG ATT ACG	*ciaD*-*ACD* construct
MEK4722	pRY111-HygR-NcoI-RV	GAC AAA CTG GGC CAT GGG ATT TAT CAT GCC TTT CTT TGT CTG TAT TCT CTC	*ciaD*-*ACD* construct

## Supplementary Materials

Supplementary materials can be found at https://www.mdpi.com/2076-2607/8/3/389/s1.
