# Supplementary material for: Molecular Dissection of the Campylobacter jejuni CadF and FlpA Virulence Proteins in Binding to Host Cell Fibronectin
Source: Microorganisms. 2020 Mar 11;8(3):389. doi: 10.3390/microorganisms8030389 (PMC7143056; doi:10.3390/microorganisms8030389)
Supplement: Supplementary file 1 [file microorganisms-08-00389-s001.pdf]

microorganisms

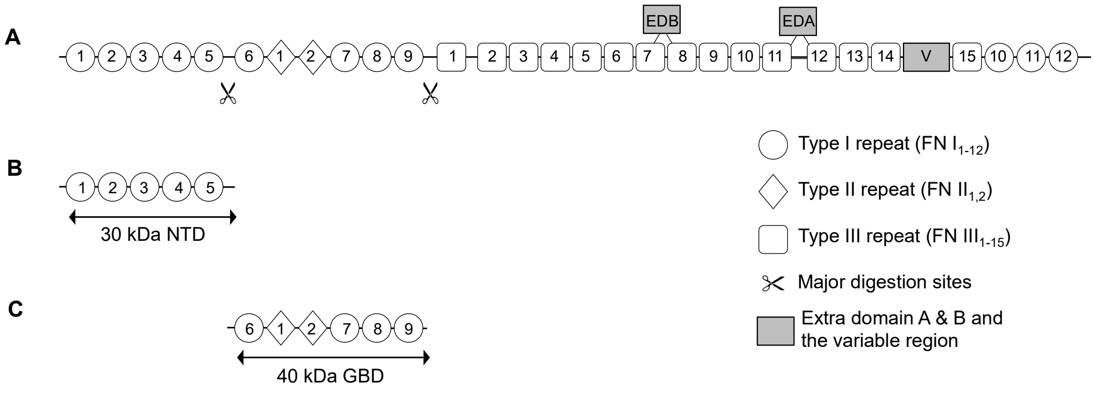

Figure S1. Schematic of fibronectin (FN) structure and FN-fragments generated by enzymatic digestion. The FN molecule is composed of multiple copies of three repeats or modules (FN I, FN II, and FN III). The structure of FN has been equated to "beads on a string." There are multiple FN isoforms that are distinguished by the presence or absence of three alternatively spliced FN repeats: extra domain A (EDA), extra domain B (EDB), and a variable region (V region). Cathepsin D digestion of FN yields an N-terminal 70-kDa fragment, and tryptic digestion of the 70-kDa fragment yields the 29–30 kDa N-terminal domain (NTD) harboring the heparin-binding domain (Panel **B**) and the 40-45 kDa NTD harboring the gelatin-binding domain (Panel **C**).

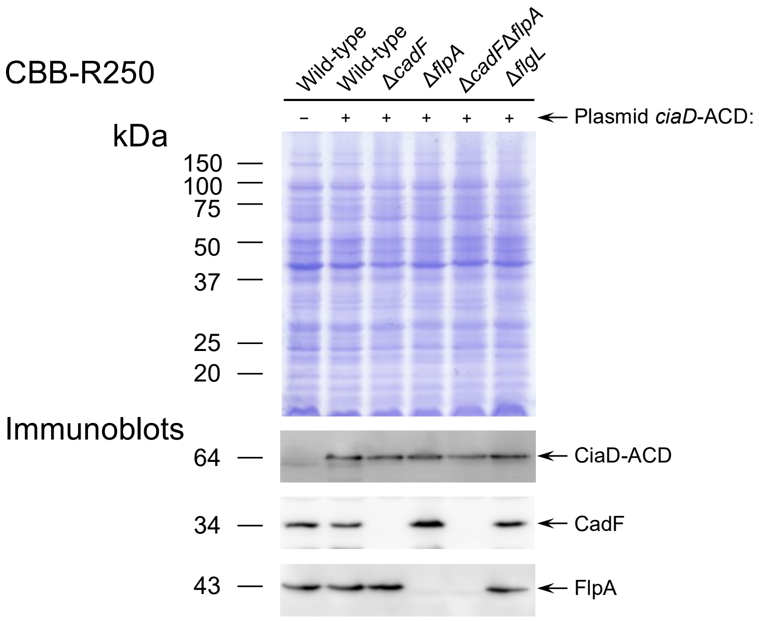

Figure S2. *C. jejuni* transformants produce the CiaD-Adenylate cyclase fusion protein (CiaD-ACD) as judged by immunoblot using α-CyaA antibody. Whole cell lysates were prepared from bacteria grown overnight in MH broth supplemented with 0.01% deoxycholate. Proteins were separated by SDS-PAGE, transferred to PVDF membranes and probed with the α-CyaA serum, α-CadF serum, and α-FlpA serum. The location of the CiaD-ACD, CadF, and FlpA proteins are highlighted (arrows). The molecular mass standards are indicated on the left (in kDa).

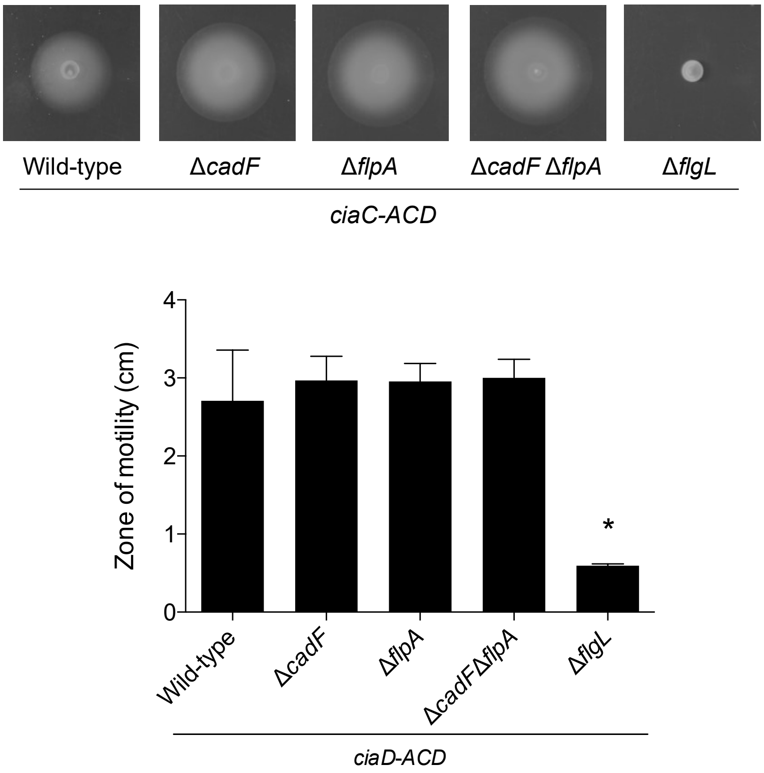

Figure S3. Transformation of the *C. jejuni* isolates with the pRY111-Hygro^{R}-P_{cysM} *ciaD-ACD* shuttle vector insert does not alter their motility. A *C. jejuni* wild-type strain, Δ*cadF* mutant, Δ*flpA* mutant, Δ*cadF* Δ*flpA* mutant, and Δ*flgL* mutant (negative control) were transformed with a shuttle vector containing the *ciaD-ACD* insert. A constant amount of the *C. jejuni* suspension was spotted onto the surface of a 0.4% MH agar plate (top panel) and the diameter of the resulting bacterial swarm was measured (bottom panel). Shown in the top panel is one representative image for each isolate. Shown in the bottom panel are the mean values (diameters) ± standard deviation of a minimum of three motility assays. The diameter of motility for the *flgL* mutant represents the size of the inoculation spot, as this isolate is non-motile.
